# 3D bioprintable Mg^2+^-incorporated hydrogels tailored for regeneration of volumetric muscle loss

**DOI:** 10.7150/thno.103677

**Published:** 2025-01-13

**Authors:** Moon Sung Kang, Jeong Min Kim, Hyo Jung Jo, Hye Jin Heo, Yun Hak Kim, Kyung Min Park, Dong-Wook Han

**Affiliations:** 1 Department of Cogno-mechatronics Engineering, Pusan National University, Busan 46241, Republic of Korea.; 2 Department of Bioengineering and Nano-bioengineering, Incheon National University, Incheon 22012, Republic of Korea.; 3 Department of Anatomy, School of Medicine, Pusan National University, Yangsan 50612, Republic of Korea.; 4 Department of Biomedical Informatics, Pusan National University, Yangsan 50612, Republic of Korea.; 5 Medical Research Institute, School of Medicine, Pusan National University, Yangsan 50612, Republic of Korea.; 6 Periodontal Disease Signaling Network Research Center and Dental and Life Science Institute, School of Dentistry, Pusan National University, Yangsan 50612, Republic of Korea.; 7 Research Center for Bio Materials & Process Development, Incheon National University, Incheon 22012, Republic of Korea.; 8 Institute of Nano-Bio Convergence, Pusan National University, Busan 46241, Republic of Korea.

**Keywords:** magnesium peroxide, 3D bioprinting, *in situ* crosslinking, volumetric muscle loss, myogenesis

## Abstract

**Rationale:** Current therapeutic approaches for volumetric muscle loss (VML) face challenges owing to limited graft availability and insufficient bioactivity. Three-dimensional (3D) bioprinting has become an alternative technology for fabricating native tissue-mimetic grafts, allowing for tailored structures and complex designs.

**Methods:** We developed an Mg^2+^-incorporated bioink composed of thiolated gelatin (GtnSH) and maleimide-conjugated gelatin (GtnMI) decorated with magnesium peroxide (MgO_2_), referred to as a GtnSH/GtnMI/MgO_2_ bioink. We designed *in situ* crosslinking between GtnSH and GtnMI to prepare cytocompatible bioink for 3D bioprinting of muscle mimetics.

**Results:** The incorporated MgO_2_ particles provided oxygen supplementation and myogenic cues. *In vitro* assays demonstrated that C2C12 myoblasts encapsulated in the GtnSH/GtnMI/MgO_2_ bioink exhibited high viability, intrinsic proliferation rate, and increased expression of key myogenic markers. *In vivo* transplantation of the 3D bioprinted GtnSH/GtnMI/MgO_2_ constructs facilitated muscle mass restoration and M2 macrophage polarization. Additionally, they downregulate the activities of CD4^+^ and CD8^+^ lymphocytes, inducing a transition from the initial inflammatory to the restoration phase.

**Conclusion:** The GtnSH/GtnMI/MgO_2_ bioink is a potential therapeutic strategy for enhancing myogenesis and skeletal muscle tissue regeneration.

## Introduction

Excessive skeletal muscle loss is often caused by traumatic injury, which leads to atrophy 1, infections 2, 3, aging, chronic diseases 4, and muscular dystrophy 5. For minor losses (< 20% volume), regeneration process can effectively restore nearly full function and muscle mass 6. However, severe damage (*e.g*., volumetric muscle loss, VML) hinders expedited regeneration, involving scarring and incomplete functional restorations 7. Current therapy for VML involves autologous muscle flap transfer 8, pharmaceutical treatment (*e.g*., anabolic steroid and myostatin inhibitors) 9, 10, and decellularized allogeneic or xenogeneic grafts 11. However, significant challenges remain, these include limited graft availability, donor site morbidity, complicated surgical handling, and various side effects such as hormonal imbalances and cardiovascular problems 12, 13.

Three-dimensional (3D) bioprinting has become prominent in newly emerging bioengineering techniques, leading to the successful fabrication of muscle mimetics and allowing for highly tailored structures and complex designs 14-16. The source materials for 3D bioprinting (*i.e.*, bioinks, a mixture of biomaterials and cells) should have mechanical and biological versatility 13, 17. They should have tunable viscoelastic properties to fabricate mechanically stable microscale cell-loaded filaments while avoiding printer nozzle blockage and maintaining proper shape stability in the cell culture environment. In addition, proper bioink candidates should recapitulate the extracellular matrix (ECM) niche to provide mechanotransductive and biochemical signaling for cell growth and myogenesis, while having reabsorbance properties to allow host-tissue integration. Hydrogels are the gold standard bioink materials because of their unique microporous structure, biocompatibility, and biodegradability 18-20. Various crosslinking methods have been introduced for hydrogels (*e.g.*, chemical crosslinking, enzymatic crosslinking, light-assisted chemical reactions, and coupling methods) to enhance their durability and make them effective scaffold materials 21. However, although 3D bioprinted muscle grafts have shown substantial potential for treating VML injuries, the limited oxygen diffusion distance (< 200 µm) leads to an insufficient nutrient supply for the cells at the center of the constructs. Therefore, the current focus is to ensure the uniform viability and maturity of embedded muscle cells.

Recent studies have shown that magnesium ions (Mg^2+^) are crucial for muscle function. The intramuscular Mg²⁺ comprises 27% of the entire body's magnesium and is crucial in skeletal muscle by supporting adenosine triphosphate (ATP) production, protein synthesis, transmembrane transport, and muscle contraction and relaxation by acting as an antagonist for Ca^2+^ channels and Ca^2+^-binding proteins 22, 23. In addition, magnesium peroxide (MgO_2_) can react with water to form Mg^2+^ and hydrogen peroxide (H_2_O_2_), leading to the long-term sustained release of oxygen 24. Providing supplemental H_2_O_2_ to damaged skeletal muscles (*i.e.*, hypoxia following trauma-related ischemia) elevates the intracellular levels of reactive oxygen and nitrogen species (RONS) to activate several signaling pathways for myogenesis 25-27. Furthermore, our previous studies demonstrated that hyperoxia-inducible hydrogels offer a regenerative niche that supports the wound healing process, suggesting the potential of MgO_2_ to promote myogenesis 28-31.

**Figure 1:**
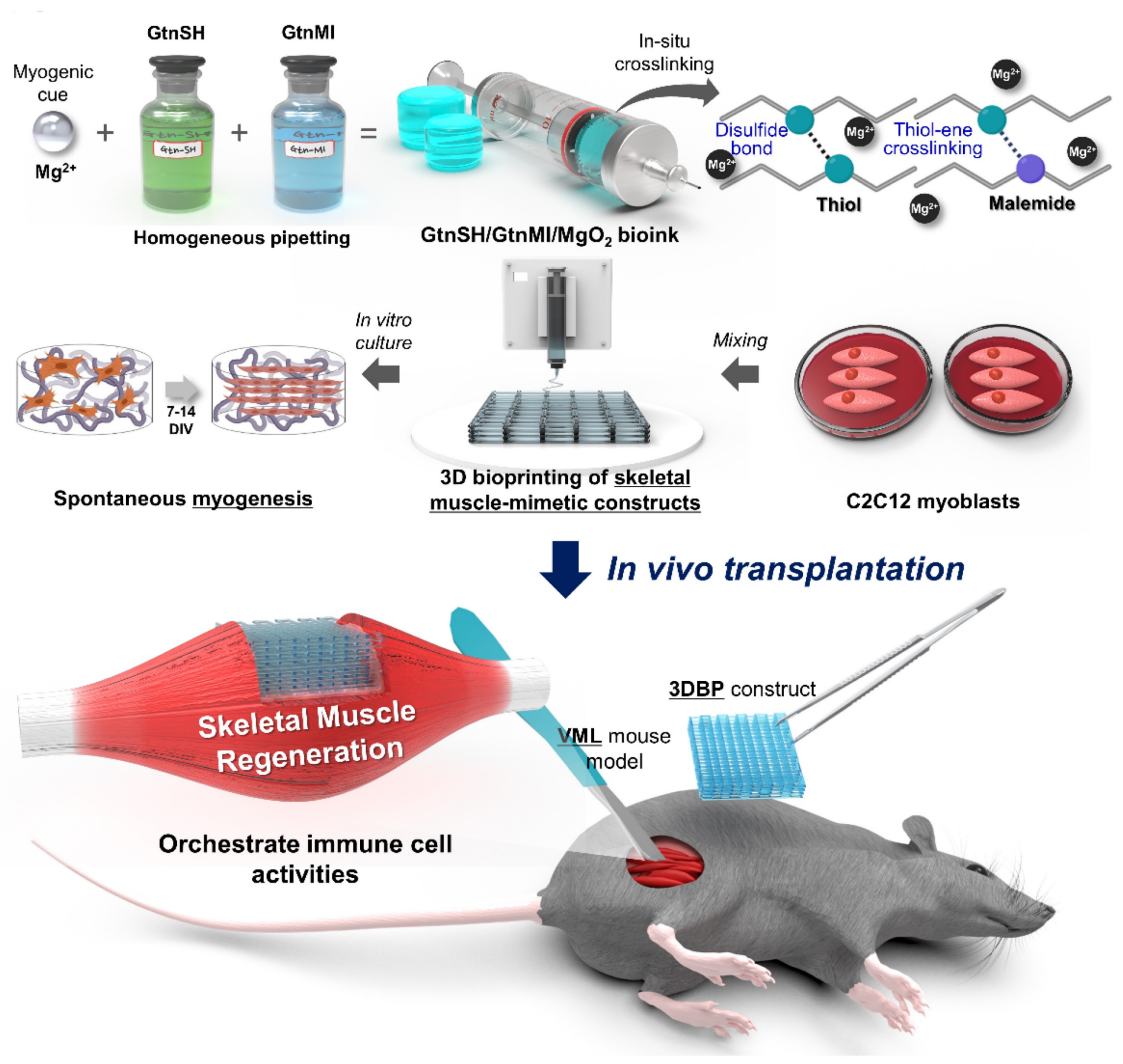
Schematic diagram of 3D bioprinting of GtnSH/GtnMI/MgO_2_ bioink and its transplantation in mouse VML models.

This study stems from previous studies that reported the myogenic capability of Mg^2+^ 22, 32-34. To the best of our knowledge, no previous reports have documented its utilization as a bioink component despite the significant roles of Mg^2+^. Moreover, the direct role of Mg^2+^ in myogenesis, both *in vitro* and *in vivo*, requires further clarification. In this study, we developed an Mg^2+^-incorporated bioink composed of thiolated gelatin (GtnSH) and maleimide-conjugated gelatin (GtnMI) decorated with magnesium peroxide (MgO_2_), referred to as the GtnSH/GtnMI/MgO_2_ bioink (**Figure 1**). A dual crosslinking strategy using GtnSH and GtnMI was designed to enable tailored *in situ* crosslinking in a cytocompatible environment. C2C12 myoblasts encapsulated in the GtnSH/GtnMI/MgO_2_ bioink exhibited high viability, proliferation, and increased expression of key myogenic markers. We also developed a mouse VML model and transplanted 3D bioprinted GtnSH/GtnMI/MgO_2_ muscle-mimetic grafts, which enhanced the restoration of motor function within seven days. The mechanisms underlying MgO_2_-induced myogenesis were further elucidated by analyzing the activity of immune cells and marker expression *in vivo*.

## Materials and Methods

### Preparation of GtnSH/GtnMI/MgO_2_ bioinks and 3D bioprinting process

GtnSH (60 mg) and GtnMI (60 mg) were added to 1 mL of Dulbecco's phosphate buffered saline (DPBS, Welgene, Daegu, Republic of Korea) and maintained in a 37 ºC water bath for 10 min to prepare bioink precursors. The 10, 20, and 30 μg mL^-1^ of MgO_2_ were added and homogeneously mixed by the vortex to prepare 3 w/v% GtnSH/GtnMI/MgO_2_ bioinks (labeled as MG0 - MG30) (**Table S1**). The detailed synthesis methods are presented in the Supplementary Information (Section 1.2.).

The 1 × 10⁶ cells mL⁻¹ C2C12 cells were incorporated into the prepared GtnSH/GtnMI/MgO_2_ bioinks and homogeneously mixed to be loaded on the 3D bioprinter (BioX, Cellink, Göteborg, Sweden). Considering the crosslinking time, the cell-loaded bioinks were directly 3D printed in pre-determined printing conditions as follows: print bed temperature of 10 °C, inner nozzle size of 580 µm, pneumatic pressure of 15 kPa, printing speed of 5 mm s^-1^, nozzle-to-print bed distance of 100 µm, and a lattice inner filling density of 40%. The 20 mm × 20 mm × 2 mm (w × l × h) sized lattices (modeled by Rhinoceros 8, McNeel, Seattle, WA) were 3D printed and maintained at room temperature (RT, 20 - 25 °C) for 10 min for stabilization. Subsequently, 3D bioprinted constructs were cultured in 6-well plates and maintained in a 37 °C, 5% CO_2_ incubator, with the media being replaced every 48 h.

### Animal test conditions and development of mouse VML model

The Animal Care Committee of Pusan National University approved animal procedures for the VML model (PNU-2024-0237). Eight-wk-old male C57BL/6 mice were kept in a barrier-protected, SPF-grade animal facility. The mice were sedated with 3% isoflurane and maintained under anesthesia with 1 - 1.5% isoflurane. To induce VML in the tibialis anterior (TA) muscle, a healthy TA muscle was injured using a 2 mm diameter biopsy punch, which is a common method for preparing a mouse VML model 35, 36. The injured area was then covered with 3D bioprinted GtnSH/GtnMI/MgO_2_ constructs, and tissue regeneration at the wound site was assessed at specific time points. The test groups were transplanted onto one side, whereas the control groups were transplanted onto the opposite TA muscles. To assess the tissue compatibility of 3D bioprinted GtnSH/GtnMI/MgO_2_ constructs, an animal study was performed using a protocol approved by the Incheon National University Institutional Animal Care and Use Committee (INU-ANIM-2022-02).

## Results and Discussion

### Cytocompatibility and myogenic capability of MgO_2_ particles

**Figure 2:**
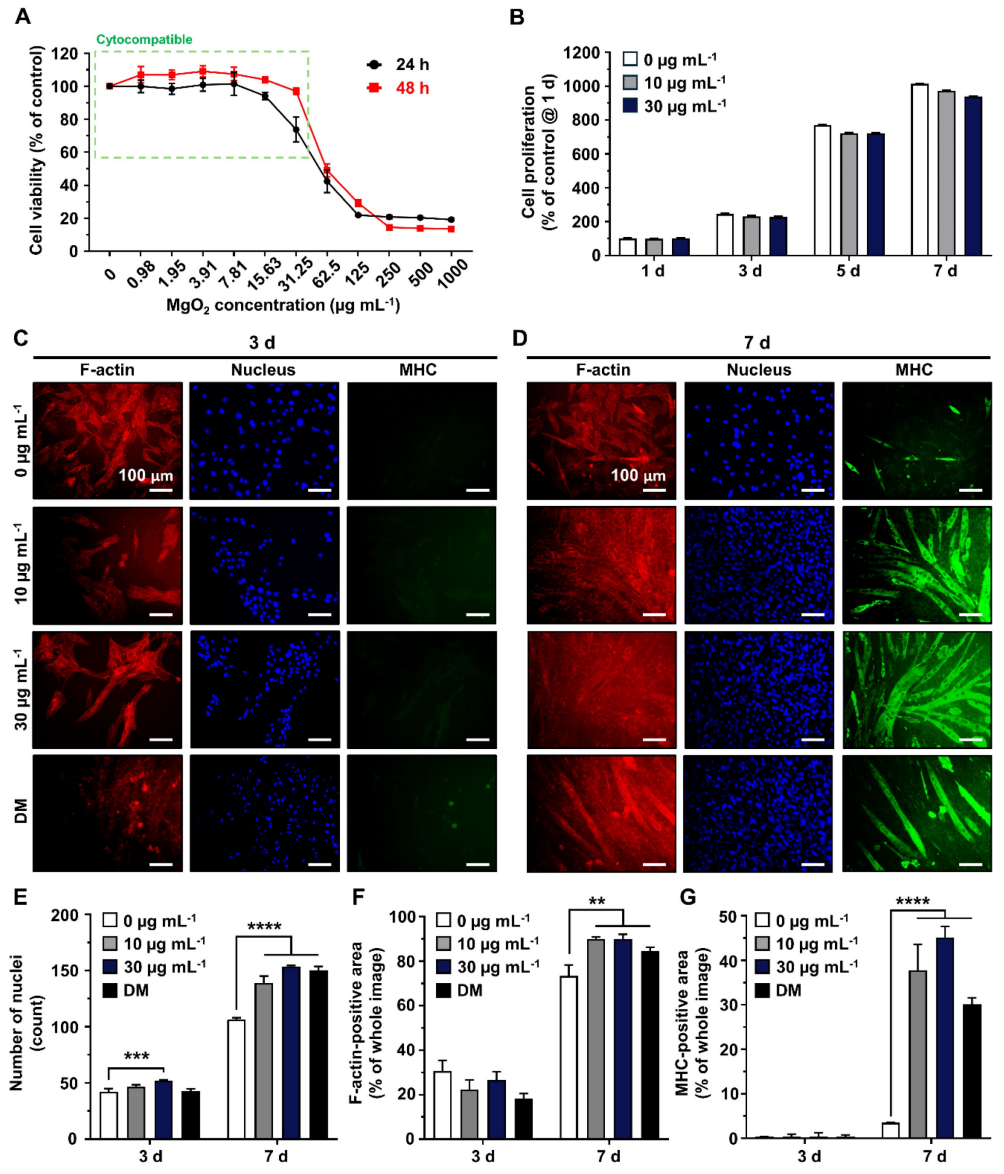
Cytocompatibility and myogenic capability of MgO_2_ particles on C2C12 cells. (A) Dose-dependent cytotoxicity of MgO_2_ particles on C2C12 cells. The green dotted square indicates the cytocompatible concentration range. (B) Cell proliferation rates of C2C12 cells during 7 d of MgO_2_ particle treatment. Immunofluorescence staining of C2C12 after (C) 3 d and (D) 7 d of MgO_2_ particle treatment. Each fluorescence channel indicates as follows: TRITC (red) for F-actin, DAPI (blue) for the nucleus, and FITC (green) for MHC. Quantification of fluorescence expression for (E) number of nuclei, (F) F-actin positive area, and (G) MHC-positive area. Asterisks indicate statistical differences between groups (**p < 0.01, ***p <0.001 and ****p < 0.0001) The scale bars indicate 100 µm for (C, D).

Most nano- and micron-scale particles exhibit cytotoxic mechanisms, including the induction of oxidative stress and mechanical damage to cellular structures and organelles 37. These harmful effects are typically mitigated in a dose- and time-dependent manner. Hence, the concentration should be optimized to avoid potential cytotoxicity and disruption of cellular behaviors 38-40. The Scanning Electron Microscopy/Energy Dispersive X-ray Spectrometer (SEM/EDS) analysis of MgO_2_ particles reveals that most are smaller than 30 µm, with no signs of aggregation and high composition of magnesium and oxygen (**Figure S1**). To optimize the concentration of MgO_2_ particles in the GtnSH/GtnMI hydrogel, they were treated with the particles (TCP)-cultured C2C12 cells (**Figure 2**). The Cell Counting Kit-8 (CCK-8) assay results showed that the viability of C2C12 cells remained above 70% after treatment with MgO_2_ at a concentration of 31.25 µg mL^-1^ (97.03% at 24 h and 73.33% at 48 h) (**Figure 2A and S2**). However, MgO_2_ exceeding 62.5 µg mL^-1^ induced significantly lowered cell viability at both 24 h (49.04%) and 48 h (42.45%), suggesting MgO_2_ ≥ 62.5 µg mL^-1^ can induce severe cytotoxicity. Subsequently, we assessed the amount of intracellular lactate dehydrogenase (LDH) in the serum that leaked from the damaged cell membrane 41. We found a similar trend in the LDH assay, showing that the MgO_2_ ≥ 62.5 µg mL^-1^ on 24 h can increase the LDH release (217.21% at 62.5 µg mL^-1^ and 400.43% at 125 µg mL^-1^) (**Figure S3**). There were no significant reducing effects of 30 µg mL^-1^ MgO_2_ on proliferation rates of C2C12 cells, suggesting 30 µg mL^-1^ can be used for further long-term cultures (**Figure 2B**). Additionally, the dichlorofluorescein diacetate (DCFDA) assay results showed that there was no significant increase of intracellular reactive oxygen species (ROS) up to 125 µg mL^-1^, further demonstrating 30 µg mL^-1^ do not induce oxidative stresses to the laden cells (**Figures S4A-B**).

To evaluate the myogenic capacity of MgO_2_ particles, 10 and 30 µg mL^-1^ MgO_2_ particles were treated on C2C12 cells, and their myogenic capability was compared with negative (w/o MgO_2_) and positive (differentiation medium, DM) controls. The myosin heavy chain (MHC), a well-known marker of early myogenesis, was stained with green fluorescence. Simultaneously, F-actin and nucleus were counterstained with red and blue fluorescence, respectively (**Figures 2C-D**) 42, 43. On 7 d, after 10 and 30 µg mL^-1^ MgO_2_ were treated, the C2C12 myoblasts were fully differentiated into the matured myotubes with a clear expression of MHC. Quantitative analysis of the fluorescence images further supported these findings. On 7 d, the number of nuclei in the 10 µg mL^-1^ (139 count) and 30 µg mL^-1^ (153 count) MgO_2_ groups was significantly (p < 0.0001) higher than the control (106 count) due to the dense clustering of multinucleated cells in the fixed field of view (**Figure 2E**). Additionally, on 7 d, the F-actin-positive area was significantly (p < 0.01) increased both in 10 µg mL^-1^ (89.84%) and 30 µg mL^-1^ (89.98%) MgO_2_ groups compared to the control (73.34%), due to the vast stretched tubular F-actin structures (**Figure 2F**). On the same day, the MHC-positive area of 10 µg mL^-1^ (37.71%) and 30 µg mL^-1^ (45.13%) MgO_2_ groups were significantly (p < 0.0001) increased compared to the control (3.52%), suggesting the MgO_2_ treatment can induce spontaneous myogenic differentiation of C2C12 myoblasts without external differentiation inducers (*e.g.*, horse serum) (**Figure 2G**).

### Physicochemical characterization of GtnSH/GtnMI/MgO_2_ bioinks

**Figure 3:**
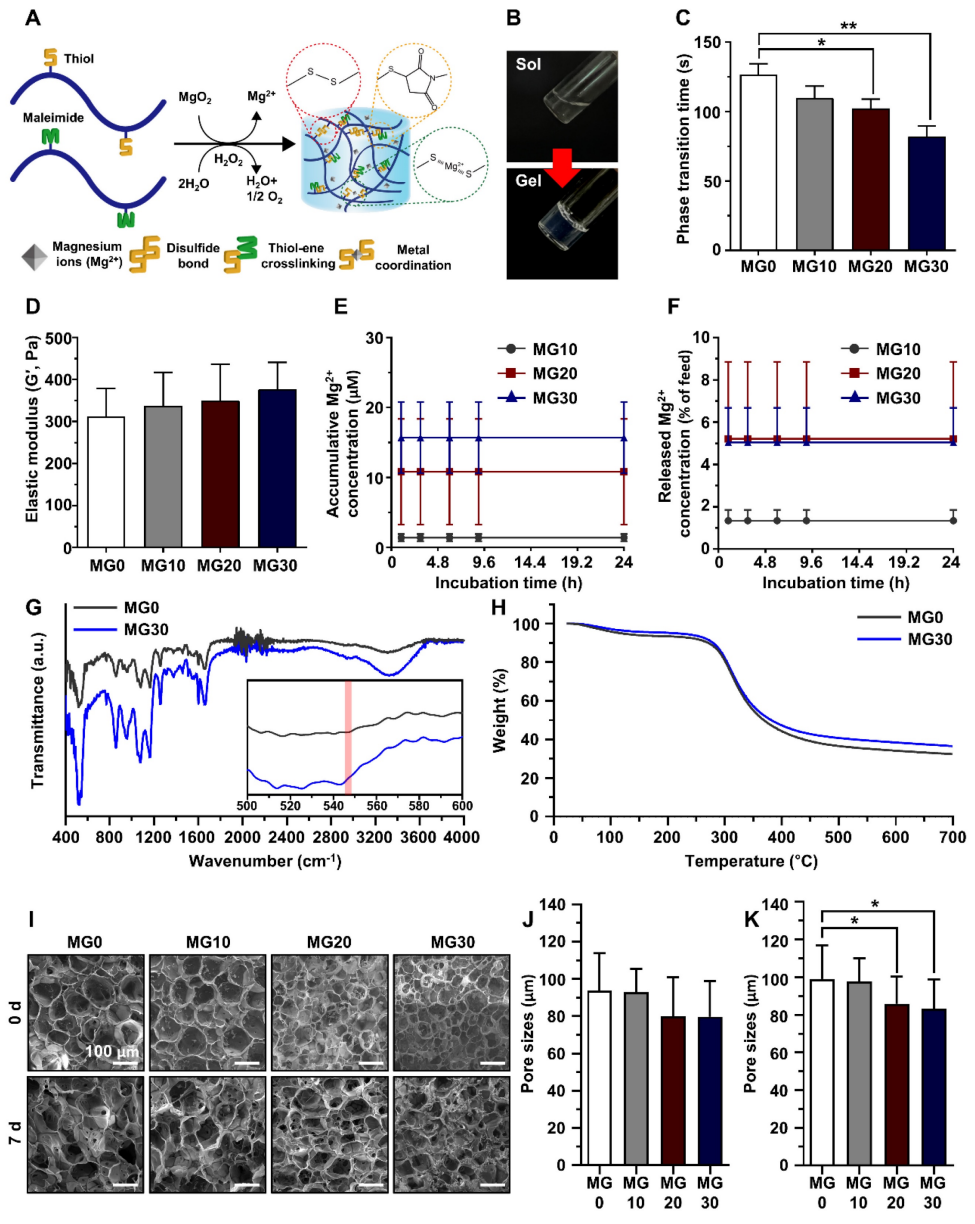
Preparation and physicochemical characterization of GtnSH/GtnMI/MgO_2_ bioinks. (A) Schematic diagram and (B) sol-gel transition of *in situ* crosslinking between functional groups within the GtnSH/GtnMI/MgO_2_ bioinks. (C) Phase transition time and (D) elastic modulus of GtnSH/GtnMI/MgO_2_ bioinks with different concentrations of MgO_2_ included. (E) Accumulated Mg^2+^ concentration released from the hydrogels and (F) released Mg^2+^ feed amount concentration in the media at each time point. (G) Fourier-transform infrared spectroscopy (FT-IR) spectra and (H) thermogravimetric analysis (TGA) graph for MG0 and MG30. The inset of (G) indicates 500 - 600 cm^-1^ wavelengths, and the red column denotes MgO_2_ specific peak at 545 cm^-1^. (I) SEM images and quantified pore sizes of cryosectioned GtnSH/GtnMI/MgO_2_ bioinks on (J) 0 d and (K) 7 d. Asterisks indicate statistical differences between groups (*p < 0.05 and **p < 0.01).

Gelatin is biocompatible, biodegradable, and bioactive, making it the backbone of most functional polymers 44. Therefore, gelatin was selected as the polymer backbone in this study. We synthesized gelatin-based polymers in which thiol and maleimide were conjugated to gelatin *via* EDC/NHS chemistry as previously reported 45. As illustrated in **Figure 3A**, we fabricated the Mg^2+^-incorporated hydrogels by mixing GtnSH, GtnMI, and MgO_2_ solutions. Hydrogels were formed *via* disulfide bonds and thio-ene reactions between the GtnSH and GtnMI polymers. **Figure 3B** shows the phase transition of the MgO_2_-mediated *in situ* crosslinkable hydrogels.

To determine the optimal phase transition time for 3D bioprinting, the hydrogel phase transition time was measured using the vial tilting method. Therefore, we found that the phase transition time decreased with increasing concentration of MgO_2_ from 127 to 92 s, with a significant decrease in MG20 and MG30 compared to MG0 (**Figure 3C**). This is because an increase in MgO_2_ concentration accelerates the crosslinking reaction. In our hydrogel system, MgO_2_ acts as a crosslinking enhancer that facilitates the formation of disulfide bonds through decomposition. This is because MgO_2_ can degrade to Mg^2+^ and H_2_O_2_ in phosphate buffers at pH 7 46, 47. The intermediate product, H_2_O_2_, creates transient oxidative stress within the polymer solution and promotes disulfide bond formation 48.

The application of hydrogels in biological environments requires adequate mechanical properties to provide three-dimensional (3D) networks for structural support and cell growth 49, 50. The prepared GtnSH/GtnMI/MgO_2_ hydrogels exhibited proper tissue adhesiveness, showing a range from 20.6 - 26.1 kPa which is stronger than the adhesive strength of FAD-approved fibrin glue (5 - 10 kPa) (**Figure S5**) 51. As shown in **Figure 3D**, the elastic modulus of the hydrogels increased from 310 to 380 Pa as the concentration of MgO_2_ increased, but the difference was not significant. This indicates that the MgO_2_ introduced into the hydrogel did not affect the mechanical strength. These results confirmed that the concentration of MgO_2_ could control the phase transition time but did not affect the mechanical strength of the hydrogel. Therefore, hydrogels with different Mg^2+^ contents but similar mechanical strengths can be prepared.

Next, the release of Mg^2+^ from the hydrogels was evaluated. We found that the higher the MgO_2_ concentrations in the hydrogels, the greater the amount of Mg^2+^ released from the matrices after 24 h in all groups (Mg10: 1.4 μM; Mg20: 10.82 μM; Mg30: 15.71 μM) (**Figure 3E**). Interestingly, over 90% of Mg^2+^ remained within the matrices (**Figure 3F**). Recently, various biomaterials that release Mg^2+^ have been reported 52-55. One of the most significant limitations of Mg^2+^-releasing biomaterials is the toxicity caused by the burst release of Mg^2+^ 56-58. Similar to other divalent cations, Mg^2+^ coordinates with various functional groups such as thiol, carboxyl, and bisphosphate 54, 55, 59. In our hydrogel system, thiol groups and various functional groups located on the gelatin backbone interacted with Mg^2+^, controlling the release of Mg^2+^. This indicates that Mg^2+^ can be present in the hydrogel matrices for an extended period and that our hydrogel can provide a constant Mg^2+^ environment.

As mentioned previously, H_2_O_2_ is generated as an intermediate byproduct during MgO_2_-mediated hydrogel formation. Although H_2_O_2_, a type of ROS, can assist in forming disulfide bonds within the hydrogel by inducing transient oxidative stress, it can cause cytotoxicity at concentrations exceeding a certain threshold. Furthermore, H_2_O_2_ exhibits cytotoxicity at concentrations exceeding 200 uM 60. We measured the released H_2_O_2_ from the hydrogel matrices. We confirmed that all groups released H_2_O_2_ for 7 d below 200 µM (MG0: 40.42 μM; MG10: 97.01 μM; MG20: 112.82 μM; MG30: 136.02 μM) (**Figure S6**). This is consistent with the previous cytotoxicity results depending on the concentration of MgO_2_ (**Figures 2A and S4**), suggesting that the concentrations of MgO_2_ used for hydrogel fabrication are within a range that does not induce cytotoxicity. In addition, the GtnSH/GtnMI/MgO_2_ bioinks did not have an oxygen-generating capacity *in vitro*, showing a consistent level of partial oxygen within the hydrogel matrices (**Figure S7**).

FT-IR was performed to analyze the crosslinking and composition of the hydrogels (**Figure 3G**). Bands of amide III (1254 cm^-1^), amide I (1632 cm^-1^), C-H_3_ (2940 cm^-1^), and O-H bonds (3320 cm^-1^) corresponding to the gelatin backbone were observed in all experimental groups 61, 62. The band around 550 cm^-1^ is an S-S bond, meaning the thiol functional groups have reacted, successfully forming a disulfide bond 63. In the MgO_2_-containing groups, a peak was formed at approximately 545 cm^-1^, corresponding to the Mg-O bond, indicating that MgO_2_ was successfully loaded during hydrogel fabrication 64, 65. The C-S-C band (1104 cm^-1^) observed in all groups is associated with the thiol-ene reaction between thiol S-H (2540 cm^-1^) and C=C (1650 cm^-1^) 66, 67. These results demonstrated that the Mg^2+^-incorporated hydrogel was successfully fabricated.

To investigate the thermal stability and decomposition behavior of the hydrogels, we performed a TGA (**Figure 3H**). The TGA curve of the fabricated gelatin-based hydrogel closely resembles that of pure gelatin. An initial weight loss of approximately 5 - 10% up to 283.6 °C corresponds to water evaporation. Subsequently, a second weight loss occurs at approximately 52 - 55% up to 524.64 °C, indicating the occurrence of thermal degradation of gelatin. As the concentration of MgO_2_ increased, less weight loss occurred at higher temperatures, which was attributed to the properties of MgO_2_ and its interaction with the gelatin matrix. MgO_2_ exhibits high thermal stability. The incorporation of MgO_2_ into the hydrogel effectively slowed the degradation of gelatin at high temperatures by acting as a heat stabilizer. In addition, the added MgO_2_ acts as a barrier by restricting the movement of molecules, including water and decomposition byproducts, within the hydrogel, resulting in slow weight loss.

The internal porous structure of hydrogels can significantly impact the penetration of surrounding tissues for effective tissue regeneration by circulating oxygen and nutrients *via* porous structure. In addition, the proteolytic degradability of hydrogels implies that they can facilitate tissue infiltration and are biodegraded during the tissue regeneration process *in situ* 49, 68. First, we evaluated the collagenase-sensitive degradation of the hydrogels using DPBS or collagenase type II. We confirmed that the Mg^2+^-incorporated hydrogels maintained their structure in DPBS or were completely degraded within 48 h by collagenase (5 µg mL^-1^) (**Figures S8A-B**). Next, we investigated the internal porous structure and the remaining Mg^2+^ within the hydrogels using SEM-EDS analysis on 0 d and 7 d (**Figure 3I**). At 0 d, all hydrogels showed porous structures with average pore sizes ranging from 76.67 to 93.85 μm, with no significant difference (**Figure 3J**). After incubation in DPBS for 7 d, we confirmed that the porous structure of hydrogels was maintained and that MG20 and MG30 exhibited a significant (p < 0.05) difference in pore size compared to MG0 (MG0, 99.10 μm; MG10, 97.94 μm; MG20, 85.88 μm; MG30, 83.29 μm; p < 0.05) (**Figure 3K**). The elemental ratio of Mg^2+^ within the hydrogel, as analyzed by EDS, revealed that the level of Mg^2+^ remained consistent between 0 d and 7 d **(Table S2)**.

This indicates that our hydrogels possess structural stability, maintaining an interconnected porous structure with a high water-swelling capacity (> 117% of the initial weight) (**Figures 3I and S8A**). As shown in **Figure S8B**, introducing a thiol or maleimide group into the gelatin backbone did not affect the degradability of gelatin. Almost all the Mg^2+^ remained within the hydrogel matrices, suggesting that the interaction between Mg^2+^ and various functional groups (*e.g.*, thiol and carboxyl groups) could incorporate Mg^2+^ within the hydrogels 59, 69. Therefore, our hydrogels can be used as scaffolds for 3D cell culture and Mg^2+^-delivery of bioactive materials by providing Mg^2+^, enabling the encapsulation or infiltration of cells, supplying nutrients, and exchanging waste metabolites both *in vitro* and *in vivo*.

The fidelity of the 3D printing (3D printability, Pr) is strongly influenced by the gelation conditions of the bioink. Under-gelation of the bioink resulted in thick filaments that induced circular holes, proper gelation resulted in rectangular holes, and over-gelation led to irregular holes (**Figure S9A**). Hence, Pr can be quantitatively assessed to determine the optimal concentration of bioink to fabricate sophisticated structures (*i.e.*, Pr > 1 indicates over-gelation, and Pr < 1 indicates under-gelation) 13. Regardless of the concentration of MgO_2_, the 3D printed constructs showed highly fine micron-sized lattices with acceptable Pr values (0.98 - 1.04) (**Figures S9B-C**).

### Growth and myogenic differentiation of C2C12 cells in the 3D bioprinted muscle mimetics

**Figure 4:**
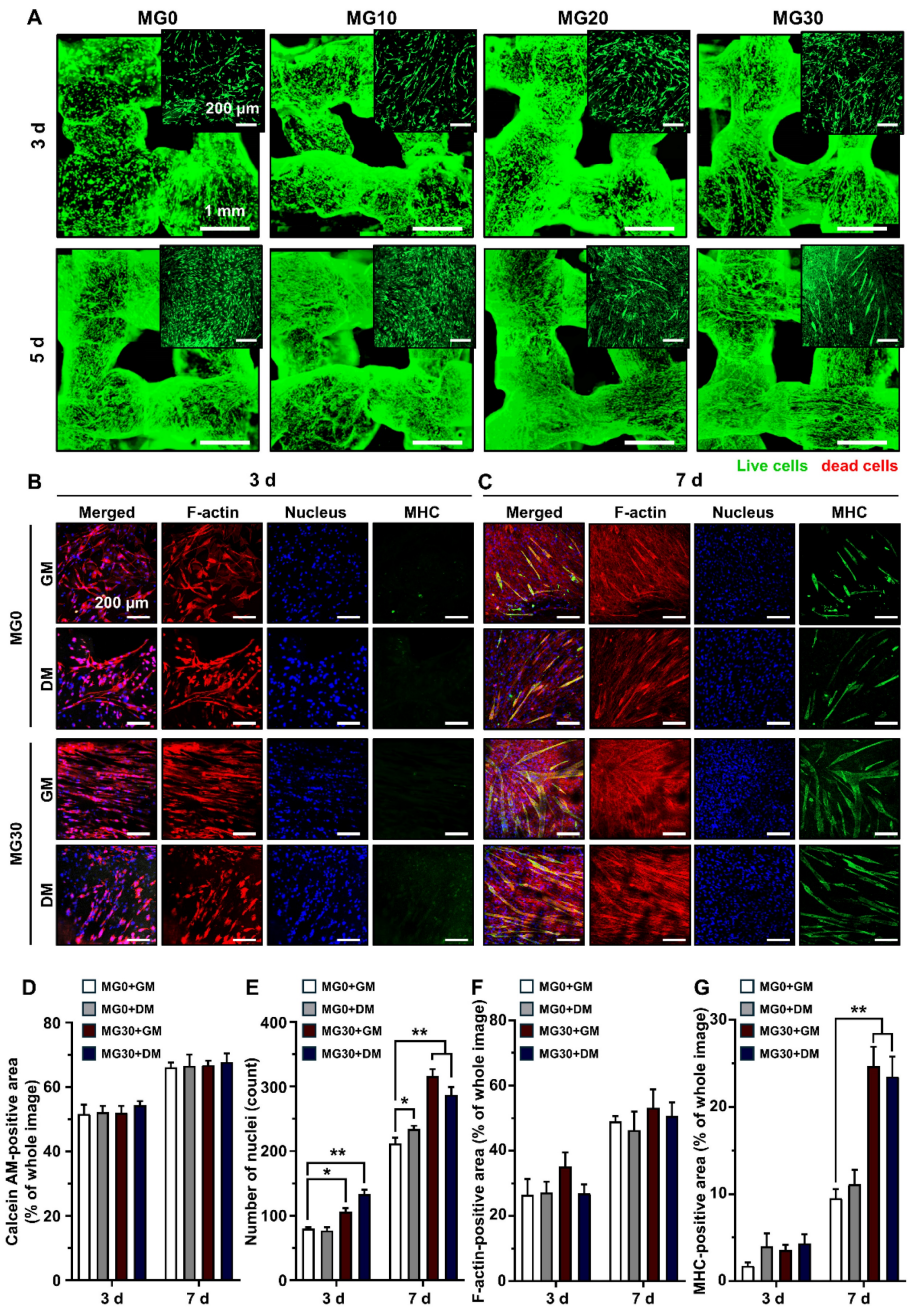
Immunofluorescence staining on C2C12 cells in the 3D bioprinted muscle mimetics. (A) Live/dead cell assay results. Live cells are stained with green (calcein AM), while red cells are stained with red (ethidium homodimer-1). (B) Immunofluorescence staining of 3D bioprinted muscle mimetics after 3 d and 7 d of 3D bioprinting. Each fluorescence channel represents the following: TRITC (red) for F-actin, DAPI (blue) for the nucleus, and FITC (green) for MHC. Quantification of (D) number of live cells by assessing calcein AM-positive area, (E) number of nuclei, (F) F-actin positive area, and (G) MHC-positive area. Scale bars indicate 1 mm for (A) and 200 µm for (A) insets and (B, C). Asterisks indicate statistical differences between groups (*p < 0.05 and **p < 0.01).

During crosslinking process, alterations in pH and temperature often affect the rheology, gelation degree, and pore size of the hydrogel, which in turn leads to reduced cell-matrix interactions and subsequent cell death 70-72. In addition, extrusion-based bioprinting generates excessive shear stress, which can rupture cell membranes and trigger apoptotic pathways 73, 74. Therefore, maintaining cell viability is a major concern in the development of bioinks and 3D printed tissue analogs 75. **Figure S10** shows that our 3D printing process did not cause significant cytotoxicity in any hydrogel group 24 h post-3D printing. The 3D bioprinting process did not cause cell death because of the relatively large nozzle size (580 nm) and low pneumatic pressure (15 kPa) used for extrusion. Rather, minimal shear stress may cause elongation of the cytoskeleton, which promotes myogenic differentiation 76, 77. We further assessed the viability of the C2C12 cells after 3 d and 5 d of 3D bioprinting (**Figure 4A**). On 3 d, MG10 - MG30 demonstrated high cell viability, comparable to that of MG0. After 5 d, MG0 - MG30 continued to show high cell viability and an increased cell population, indicating that incorporating MgO_2_ into the GtnSH/GtnMI hydrogel did not impede long-term cell viability and proliferation (**Figure 4D**). The C2C12 cells were maintained in a healthy state owing to the mild crosslinking conditions of the GtnSH/GtnMI hydrogel and the cytocompatible and cell-adhesive niche provided by gelatin.

Subsequently, we observed the morphology and expression of myogenic markers in the 3D bioprinted muscle mimetics (**Figures 4B-C**). On 3 d, the MG30 group exhibited more elongated cytoskeletal structures than the MG0 group, initiating cell fusion and forming early myotubes. On 7 d, MG30 exhibited higher MHC expression than MG0 and thicker and more mature myotube morphology. On 7 d, the number of nuclei was significantly higher in the MG30+GM (316 nuclei, p < 0.01) and MG30+DM groups (287 nuclei, p < 0.01) than in the MG0+GM (212 nuclei) and MG30+DM groups (234 nuclei) (**Figure 4E**). No significant differences were observed between the groups in the F-actin-positive area because the cells reached full confluence within 7 d (**Figure 4F**). On 7 d, MHC expression was highly increased in MG30+GM (24.71%, p < 0.01) and MG30+DM (23.49%, p < 0.01) compared to MG0+GM (9.52%), and even exceeded MG0+DM (11.12%) (**Figure 4G**). On the same day, the average myotube lengths of MG30+GM (549.61 µm, p < 0.01) and MG30+DM (660.55 µm, p < 0.0001) were significantly longer than those of MG0+GM (219.33 µm) and MG0+DM (274.55 µm) (**Figure S11A**). The maturation index was significantly higher in the MG30+GM (67.69%, p < 0.0001) and MG30+DM groups (76.25%, p < 0.0001) than in the MG0+GM (0%) and MG30+DM groups (25%) (**Figure S11B**). Notably, fusion index was most highly expressed in the MG30+GM (33.09%, p < 0.0001) group, compared to the MG0+GM (4.69%), MG0+DM (6.22%), and MG30+GM groups (14.23%) (**Figure S11C**). On 7 d, the expression of later myogenesis and maturation markers were evaluated (**Figure S12 and S13**). The MG30+GM and MG30+DM showed higher expression of dystrophin and desmin, compared to GM0+GM and GM0+DM, suggesting the incorporation of MgO_2_ in the hydrogel promote the structural integrity and contractile forces of the myotubes 78, 79. Based on these findings, we conclude that MgO_2_ promotes myogenesis in C2C12 cells, facilitating the expression of key markers in the process.

**Figure 5:**
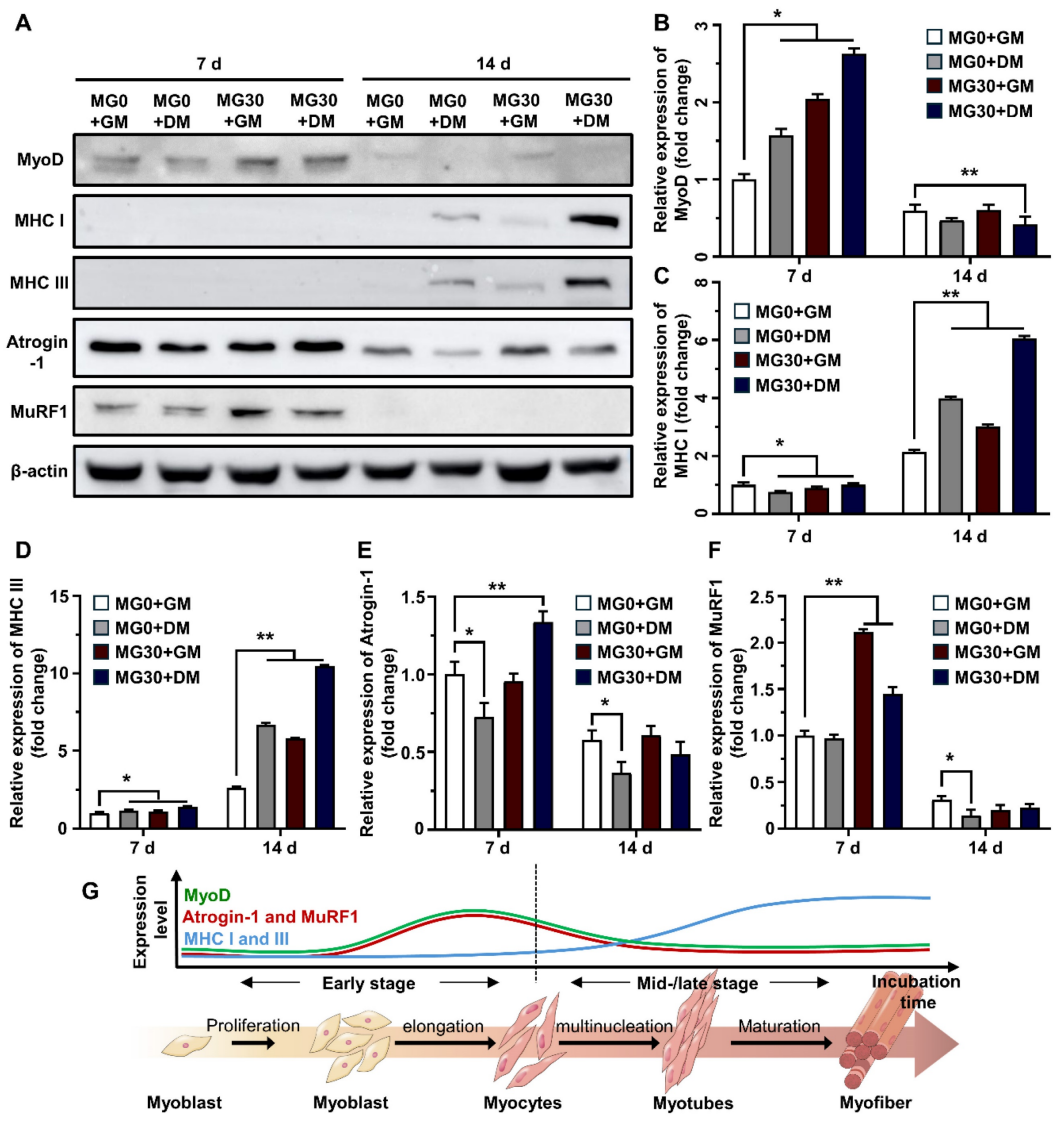
Western blotting on C2C12 cells in 3D bioprinted muscle mimetics. (A) Digital images of SDS-PAGE. Relative expression of (B) MyoD, (C) MHC I, (D) MHC III, (E) Atrogin-1, and (F) MuRF1. (G) Schematic diagram of expression levels of myogenic markers in each differentiation stage. Asterisks indicate significant differences between groups (*p < 0.05 and **p < 0.01).

To assess the role of MgO_2_ in the myogenesis of C2C12 cells within the 3D bioprinted muscle mimetics, we assessed the protein expression of myogenic markers (**Figure 5A**). MyoD is highly expressed in undifferentiated myoblasts and initiates the differentiation process by binding to the regulatory elements of muscle-specific genes, switching from a repressive to a permissive role in gene expression 80. **Figure 5B** shows that MyoD expression in MG30+GM (2.04-fold) was more significant than that in MG0+DM (1.57-fold), suggesting that MgO_2_ promotes higher MyoD levels than DM, promoting the early stages of myogenesis. The reduced expression at 14 d in the Mg30 groups could indicate that their initial role is completed and other myogenic factors become more prominent in driving the mid-terminal stages of myogenesis. MHC types I and III, which are representative myogenic markers 81, 82, were significantly increased in the MG30+GM (3.01- and 5.78-fold, respectively) and MG30+DM (6.05- and 10.48-fold, respectively) groups on 14 d (**Figures 5C-D**). Atrogin-1 and Muscle-specific RING finger protein 1 (MuRF1) are atrophy-related proteins typically upregulated during muscle degradation 83. On 7 d, the Atrogin-1 and MuRF1 levels were elevated in the MG30+GM (0.95-fold and 2.11-fold, respectively) and MG30+DM (1.34-fold and 1.45-fold, respectively) groups (**Figures 5E-F**). This can be explained by the H_2_O_2_ supplementation of MgO_2_ particles, which activates FoxO1/MuRF1/atrogin-1 signaling pathways to initiate early myogenic signals 84. Additionally, increased levels of MyoD and MHC provide positive feedback, which enhances the expression of MuRF1 and atrogin-1 during the early stages of myogenesis 85-87. However, the expression levels of Atrogin-1 and MuRF1 decreased to a level comparable to that in the MG0 group, suggesting that MgO_2_ did not have prolonged muscle atrophic effects.

Previous studies demonstrated the role of Mg^2+^ in myogenesis and muscle function. Furutani *et al.* reported that Mg^2+^ deficiency can negatively affect the early stages of myogenesis, leading to compromised myotube maturation and function by inducing oxidative stress and altering the expression of MyoD and myogenin 32. Additionally, Liu *et al.* reported that Mg^2+^ stimulates the mTOR pathway to upregulate myogenic markers and myotube hypertrophy to conserve muscle mass and strength 22. Zocchi *et al.* reported the essential role of Mg^2+^ in myogenesis, showing that a low Mg^2+^ concentration induces a significant reduction in myotube thickness and the accompanying downregulation of MHC, myogenin (MyoG), and myomixer 34. Maradze *et al.* suggested that synchronized activities between Mg^2+^ and Ca^2+^ are crucial in myotube development and that their optimum concentration ratio should be tailored 88. Another study by Cui *et al.* clearly showed that combined therapy with low-magnitude high-frequency vibration and Mg^2+^ supplementation activates the PI3k/Akt/mTOR pathway to enhance the population of CD206^+^ M2 macrophages 33. Based on our findings and those of previous studies, it is evident that MgO_2_ particles in the GtnSH/GtnMI hydrogel serve as powerful myogenesis promoters by modulating key myogenic inhibitors and enhancing the synthesis of structural proteins at various stages of differentiation (**Figure 5G**).

### *In vivo* transplantation of 3D bioprinted muscle mimetics in mouse VML models

**Figure 6:**
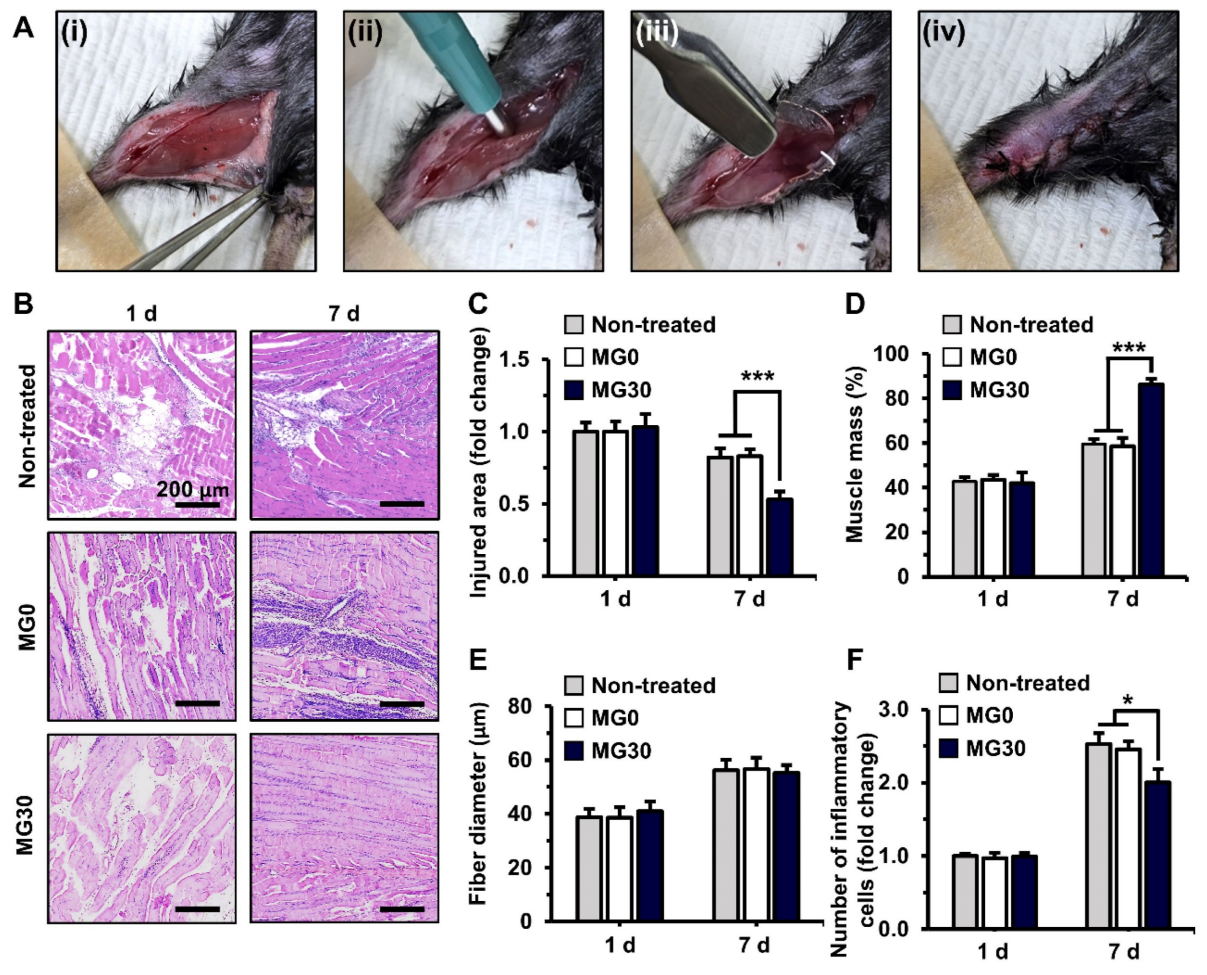
*In vivo* transplantation of 3D bioprinted GtnSH/GtnMI/MgO_2_ constructs. (A) 2 mm biopsy punch induced muscle loss in mice. (i-iv) Digital images of the surgical process. (B) H&E staining of GtnSH/GtnMI/MgO_2_-transplated TA muscle biopsy. Quantification of (C) injured area, (D) muscle mass, (E) fiber diameter, and (F) number of inflammatory cells. Scale bars indicate 200 µm for (B). Asterisks indicate significant differences between groups (*p < 0.05 and ***p < 0.001).

To induce the VML model, a 2 mm diameter biopsy punch was applied to the mouse TA muscles, resulting in the removal of 25.58% of the muscle mass by weight, which is consistent with the standard VML ratio (> 20%) (**Figure S14**) 89. The 3D bioprinted GtnSH/GtnMI/MgO_2_ constructs were transplanted into the VML of mouse TA muscles (**Figure 6A**). We examined the impact of hydrogel degradation products on major organs 7 d and 28 d after implanting in subcutaneous tissue on the back of mice. The implanted hydrogels demonstrated *in vivo* stability and proteolytic degradability, showing a time-dependent degradation of hydrogels (**Figures S15A-C**). The histological analysis for essential organs (*i.e.*, heart, kidney, liver, lung, and spleen) in the MG10 - MG30 groups revealed minimal abnormalities or lesions, nearly comparable to the control group at both 7 d and 28 d (**Figures S15D-E**). Moreover, the implanted hydrogels exhibited comparable levels of infiltrated host cells and macrophages within hydrogel matrices undergoing local foreign body reaction with matrices degradation (**Figures S16A-E**). These results further confirmed that our hydrogels have excellent tissue compatibility, suggesting that they induce minimal chronic toxicity because of their rapid bodily clearance 90, 91.

Subsequently, the extent of muscle regeneration was evaluated for over 7 d (**Figure 6B**). On 7 d, MG30 showed a significant (0.53-fold, p < 0.001) decrease in the injured area compared with the non-treated (0.82-fold) and MG0 (0.83-fold) groups, suggesting enhanced muscle recovery (**Figure 6C**). On the same day, the muscle mass in the regenerated region was significantly higher in the MG30 group (86.4%, p < 0.001) than in the untreated (59.6%) and MG0 (58.6%) groups (**Figure 6D**). Notably, muscle fiber diameter was similar between the groups on 1 d and 7 d, indicating that the increased muscle mass resulted from an increased number of muscle fibers (**Figure 6E**). On 1 d, the number of immune cells was similar between groups (**Figure 6F**). However, on 7 d, the number of immune cells in the MG30 group was significantly decreased (2.00-fold, p < 0.05) compared to that in the non-treated (2.53-fold) and MG0 groups (2.45-fold). The reduced immune cell presence at 7 d suggests that the initial inflammatory response was completed, leading to an efficient transition from the inflammatory to the regeneration phase 92, 93. Based on these results, the myogenic effects of MgO_2_ were clearly distinguished from those of the GtnSH/GtnMI hydrogels, which facilitated initial tissue regeneration.

**Figure 7:**
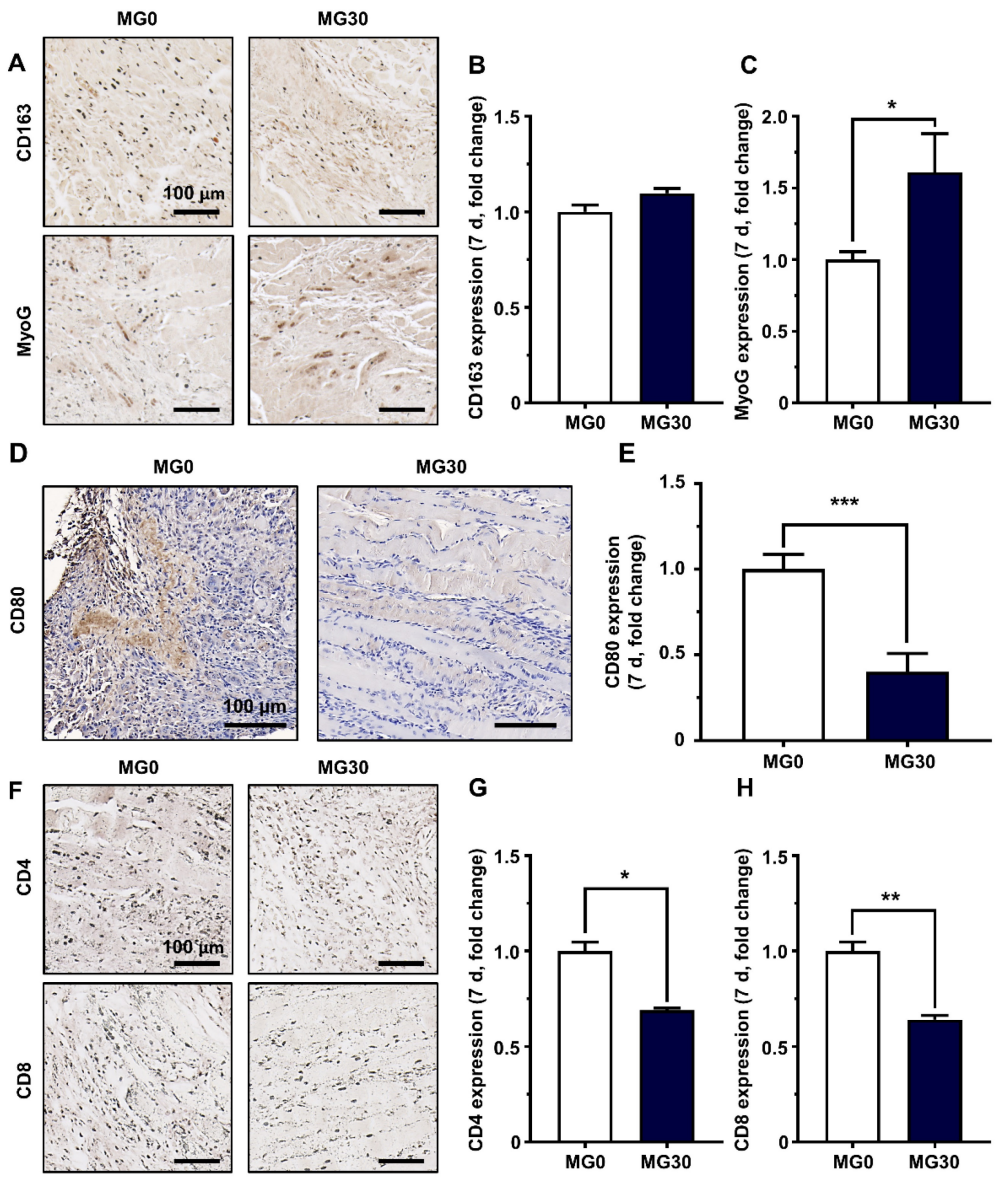
Immunohistochemical analysis on GtnSH/GtnMI/MgO_2_-transplanted TA muscle biopsy. (A) Immunohistological images and quantified expression of (B) M2 macrophage marker CD163 and (C) early myogenic marker MyoG on 7 d. (D) Immunohistological images and (E) quantified expression of M1 macrophage marker CD80 on 7 d. (F) Immunohistological images and quantified expression of T cell markers (G) CD4 and (H) CD8 on 7 d. Scale bars indicate 100 µm. Asterisks indicate significant differences between groups (*p < 0.05, **p < 0.01, and ***p < 0.001).

We further assessed the activity of each immune cell by staining for specific markers. Macrophages are crucial for inflammation and tissue remodeling after injury. Initially, pro-inflammatory M1 macrophages promote debris clearance, satellite cell proliferation, and fibrosis regulation 94. Furthermore, anti-inflammatory M2 macrophages stimulate myogenesis and angiogenesis, with their polarization supported by myogenic precursor cells, endothelial cells, and fibroadipogenic progenitors 94. Recent studies have highlighted the crucial role of macrophages in acute skeletal muscle injury repair, emphasizing that the transition to M2 phenotypes is essential for achieving functional muscle regeneration 93, 95, 96. The expression of CD163, a representative M2 macrophage, was slightly increased compared to that in the control group (**Figures 7A-B**). In contrast, CD80 expression, a marker of M1 macrophages, was significantly reduced in the MG30 group (0.40-fold, p < 0.001) compared to that in the MG0 group, indicating that MgO_2_ within the hydrogel facilitated the phenotype shift toward M2 macrophages (**Figures 7D-E**) 97. Therefore, MyoG expression was significantly increased in MG30 (1.61-fold, p < 0.05) compared with that in MG0, suggesting increased fusion into early myotubes (**Figures 7A-C**) 98.

Additionally, we assessed the expression of CD4 and CD8 in T cells to evaluate their activities (**Figure 7F**). The expression levels of CD4 and CD8 in MG30 were significantly decreased (0.69-fold, p < 0.05, and 0.64-fold, p < 0.01) compared to those in MG0 (**Figures 7G-H**). During the initial inflammatory phase, CD4^+^ T cells promote arteriogenesis and enhance skeletal muscle regeneration by boosting inflammation, activating and polarizing macrophages, and regulating their regenerative functions 99. Simultaneously, CD8^+^ T cells infiltrate the damaged muscle matrix and remodel myoblast proliferation by accelerating the secretion of the proinflammatory chemokine MCP-1/CCL2 100. Based on decreased expression of CD4+ and CD8+ T cells in MG30 groups, we further confirmed that MgO2 facilitate phase transition from inflammatory to regenerative phase. Finally, the restoration of motor function in the MG30-transplanted mice was estimated using a grip strength test (**Figure S17A**). There were no significant differences between the groups at 1 d and 3 d post-transplantation (**Figure S17B**). However, at 5 d and 7 d post-transplantation, pulling forces were significantly (p < 0.0001 and p < 0.001) increased at MG30 compared to MG0. In detail, MG30 showed 0.47 N (5 d) and 0.56 N (7 d) of pulling forces, while MG0 showed 0.37 N (5 d) and 0.42 (7 d), suggesting the restoration of motor function after MgO_2_ accelerated VML.

Our findings are consistent with those of previous studies, highlighting the role of Mg^2+^ in modulating immune responses during skeletal muscle regeneration. Liang *et al.* demonstrated that Mg^2+^ modulates the immune responses by decreasing the secretion of type 2 CD4^+^ T lymphocyte cytokines, including interleukin-5 (IL-5), IL-13, and interferon-γ (IFN-γ) 101. Furthermore, Diao *et al.* revealed that sufficient levels of free intracellular Mg^2+^ should be maintained to prevent the defective expression of programmed cell death 1 (PD-1) and NK activating receptor (NKG2D) in CD8^+^ T cells 102. Bessa-Gonçalves demonstrated that the Mg promotes M2 macrophage polarization (enhanced CD163 expression) by reducing NF-κB p65 nuclear translocation, thereby down-regulating the production of pro-inflammatory mediators (*e.g.,* IFNγ, IL-12, TNF-⍺, IP-10) 103. Hu *et al.* reported that Mg^2+^ promotes M2 macrophage polarization and down-regulate lipopolysaccharide (LPS) and interferon-γ (IFN-γ)-derived inflammatory reactions, which resulted in enhanced differentiation of mesenchymal stem cells (MSCs) 104. These findings show that our prepared GtnSH/GtnMI/MgO_2_ bioinks (especially MG30) facilitated M2 macrophage phenotype transition with the downregulation of CD4^+^ and CD8^+^ T cells, which finally induced rapid progression to the regenerative phase, resulting in an increased wound closure rate and muscle mass restoration (**Figure S18**).

## Conclusion

Proof-of-concept studies delving into living cell responses with bioink niche are essential for developing effective grafts that can be implemented in clinical applications. The GtnSH/GtnMI/MgO_2_ bioinks provide biophysical cues and biochemical signals that stimulate intrinsic cellular behaviors. In our study, the GtnSH/GtnMI/MgO_2_ bioinks provided a proper mechanical stimulus (*i.e.,* RGD peptide) for enhanced cell-matrix and intercellular interactions. Physicochemical characterization of the GtnSH/GtnMI/MgO_2_ bioinks indicated that they were well synthesized, as designed in our scheme. The incorporation of MgO_2_ improved the structural properties of the hydrogel without compromising its mechanical strength and preventing the initial burst of Mg^2+^ and H_2_O_2_. Consequently, the C2C12 myoblasts laden in the GtnSH/GtnMI/MgO_2_ bioinks showed high viability and spontaneous myogenesis with the enhanced expression of myogenic markers. *In vivo* analysis demonstrated that the VML-transplanted 3D bioprinted GtnSH/GtnMI/MgO_2_ constructs enhanced muscle mass restoration and significantly promoted muscle fiber regeneration and motor function recovery. Immunohistochemical analysis revealed increased M2 macrophage polarization and decreased numbers of CD4^+^ and CD8^+^ T lymphocytes, indicating that MgO_2_ possesses both myogenic and immunomodulatory properties. The unique myogenic and immunomodulatory effects of the GtnSH/GtnMI/MgO_2_ bioinks were compared with those of previously reported studies (**Table S3**). Our GtnSH/GtnMI/MgO_2_ bioinks target the early transition from the inflammatory phase to the regeneration phase and boost early myogenesis, which is anticipated to accelerate the overall VML restoration process 105, 106.

Recent advances in 3D bioprinting have suggested its potential for personalized grafts, contributing to paradigm shifts in current medicine. However, 3D bioprinting technology is still in the early stages of clinical translation, necessitating additional investigations to bridge this gap. Comprehensive toxicological assessments would be required *in vitro* and *in vivo*, along with a detailed analysis of the mechanism of action at the genetic level, pharmacokinetics, organ distribution, interactions, and long-term safety. Additionally, consistent regulatory guidelines (*e.g.*, sterility, protocol standardization, and storage conditions) are necessary to ensure safe utilization. In summary, further research and exploration of MgO_2_ and 3D bioprinting will deepen our understanding and reveal new opportunities for its clinical applications, which will ultimately revolutionize VML treatment.

## Supplementary Material

Supplementary methods, figures and tables.
