# Supplementary material for: 3D bioprintable Mg2+-incorporated hydrogels tailored for regeneration of volumetric muscle loss
Source: Theranostics. 2025 Jan 13;15(6):2185–200. doi: 10.7150/thno.103677 (PMC11840723; doi:10.7150/thno.103677)
Supplement: Supplementary file 1 — Supplementary methods, figures and tables. [file thnov15p2185s1.pdf]

Supplementary information for

3D bioprintable Mg^{2+}-incorporated hydrogels tailored for regeneration of volumetric muscle loss

Supplementary Materials and Methods

Cell culture environment

C2C12 murine myoblast cells were purchased from the American Type Culture Collection (ATCC, Rockville, MD). The cells were routinely cultured in a growth medium (GM) composed of Dulbecco's Modified Eagle Medium (DMEM, Welgene, Daegu, Republic of Korea) supplemented with 10 v/v% fetal bovine serum (FBS, Welgene) and 1 v/v% antibiotic-antimycotic solution (Abs, 10,000 units of penicillin, 10 mg of streptomycin, and 25 μg of amphotericin B mL^{-1}). As a positive control, the cells were cultured in a differentiation medium (DM) of DMEM supplemented with 2 v/v% horse serum (Welgene). The culture media were changed every 48 h, the cells were subcultured in Trypsin-EDTA solution (Welgene) at 80% confluency, and they were in a 37 ℃ humidified environment with 5% CO_2. The printed constructs (1×10^6 cells mL^{-1} in an approximately 100 – 200 μL bioink) were submerged in GM or DM for predetermined culture periods.

Preparation of GtnSH/GtnMI/MgO_2 bioinks

Materials for preparation of GtnSH/GtnMI/MgO_2 bioinks

For the synthesis of bioinks composed of thiolated gelatin (GtnSH), maleimide-conjugated gelatin (GtnMI), and MgO_2 (GtnSH/GtnMI/MgO_2 bioinks), gelatin (type A from porcine skin), cystamine dihydrochloride, 1-Ethyl-3-(3-dimethyl aminopropyl) carbodiimide hydrochloride (EDC), N-hydroxy succinimide (NHS), DL-dithiothreitol (DTT), 6-maleimidohexanoic acid (MHA), and magnesium peroxide complex (MgO_2 • xMgO), and collagenase type II (from clostridium histolyticum) were purchased from Sigma-Aldrich (St. Louis, MO). 1N hydrochloric acid (HCl) solution was supplied by Daejung (Siheung, Gyeonggi, Republic of Korea). The dialysis membrane bag (molecular weight cutoff = 3,500 Da) was obtained from Spectrum Laboratories (Ranchi Dominguez, CA). Ca^{2+}/Mg^{2+}-free Dulbecco's phosphate-buffered saline (DPBS) was provided by Welgene (Daegu, Republic of Korea). 5,5'-Dithio-bis(2-nitro benzoic acid) (Ellman's reagent) was obtained from Thermofisher Scientific (Waltham, MA). Hydrogen peroxide (H_2O_2, 30 w/v% in H_2O) was purchased by Junsei Chemical (Tokyo, Japan).

Synthesis and characterization of thiolated gelatin

As previously reported, the thiolated gelatin was synthesized by introducing thiol groups into a gelatin backbone via EDC/NHS chemistry [1]. Briefly, 250 mg of gelatin was dissolved in 125 mL of deionized (DI) water at 40 °C. Cystamine dihydrochloride (1.0 mmol) was dissolved in 2.5 mL DI water and added to a gelatin solution. The mixing was conducted for 10 min at 40 °C. EDC (0.5 mmol) and NHS (0.5 mmol) were dissolved in 2.5 mL of DI water and added to the gelatin/cystamine dihydrochloride solution sequentially. The reaction was performed for 2 h at 40 °C. After the EDC/NHS reaction, we added the DTT solution (2.0 mmol) dissolved in 5 mL of DI water and reacted for 24 h. After the cleavage reaction using DTT, the solution

was sequentially dialyzed against 5 mM HCl solution for 36 h and 1 mM HCl solution for 24 h using a dialysis membrane bag. This process was performed to remove byproducts of EDC/NHS reaction, such as remaining DTT molecules and unconjugated cystamine dihydrochloride molecules. After dialysis, we obtained the GtnSH polymer by freezing the polymer at –80 °C in a deep freezer, followed by a lyophilization process.

We investigated the thiol contents of the synthesized GtnSH using Ellman's assay according to the manufacturer's instructions. Briefly, 100 μL of GtnSH polymer solution and cysteine solution (1 mg mL^{-1} in DI water) were reacted with 100 μL of Ellman's reagent for 20 min. After the reaction, we measured absorbance at 405 nm wavelength using a microplate reader (Multiskan EX, Thermofisher Scientific). The thiol contents were calculated from a cysteine standard curve at known cysteine concentrations (1 – 40 μg mL^{-1})

Synthesis and characterization of GtnMI

According to our previous report, the GtnMI was synthesized by introducing maleimide groups into a gelatin backbone via EDC/NHS chemistry [1]. Briefly, 250 mg of gelatin was dissolved in 50 mL of DPBS at 40 °C. The MHA (1.0 mmol) was dissolved in 120 mL of DPBS and added to a gelatin solution. We mixed the solution for 10 min at 40 °C. EDC (1.2 mmol) and NHS (1.4 mmol) were dissolved in 5 mL of DPBS and added to gelatin/MHA solution sequentially. The reaction was conducted for 2 h. After the EDC/NHC reaction, the solution was transferred to a dialysis membrane bag (molecular weight cutoff = 3,500 Da). The dialysis was performed for 72 h to remove unconjugated MHA molecules and byproducts of the EDC/NHS reaction. After dialysis, we obtained the GtnMI polymer by freezing the polymer at –80 °C in a deep freezer, followed by a lyophilization process.

We investigated the maleimide contents of the synthesized GtnMI using Ellman's assay according to the manufacturer's instructions. Briefly, 50 μL of GtnMI solution and MHA solution (1 mg mL^{-1} in DIW) were first reacted with cysteine solution for 10 min, and secondly reacted with 50 μL of Ellman's reagent for 20 min. After the reaction, we measured absorbance at 405 nm wavelength using a microplate reader (Multiskan EX). The maleimide contents were calculated from a maleimide standard curve at known maleimide concentrations (2.5 – 40 μg mL^{-1})

Physicochemical characterizations of GtnSH/GtnMI/MgO_2 bioinks

Rheological analysis of GtnSH/GtnMI/MgO_2 bioinks

The phase transition time was performed using the *vial-tilting* method. The sol-gel transition test was measured until the solution was not flowing. The elastic modulus (G′) of the Mg^{2+}-incorporated hydrogels was measured using a rheometric fluid spectrometer (DHR-1, TA Instruments, New Castle, DE) in an oscillatory mode [2-4]. The 200 μL of hydrogels were loaded on the middle of the plate. We performed dynamic time weeps on the samples using a 20 mm parallel plate depending on the MgO_2 concentration (strain = 0.1%, frequency = 0.1 Hz, at 37 ℃). The plates were covered by a solvent trap to avoid solvent evaporation. The adhesive strength of GtnSH/GtnMI/MgO_2 bioinks were measured by universal testing machine (UTM, UNITEST M1, TEST ONE, Busan, Republic of Korea) equipped with a load cell sensor (LCK1205-K010) according to the modified ASTM standard F2255-05 method. The 50 μL of hydrogel was treated on decellularized porcine skin and the adhesive strength was measured after the stabilization for 15 min.

***In vitro* Mg^{2+} and H_2O_2 release study**

To investigate the amount of released Mg^{2+} and H_2O_2 from hydrogels, we analyzed magnesium colorimetric assay and Cu(II)-neocuproine assay. The 20 μL of Mg^{2+}-incorporated hydrogels was incubated in 500 μL of Ca^{2+}/Mg^{2+}-free DPBS at 37 °C. We collected the DPBS at each preset time point (0.5, 1, 3, 6, 9, 24, 72, 120, and 168 h) and refilled the same volume of fresh DPBS. Before proceeding with the detection assay, all samples were stored at –20 °C.

A magnesium colorimetric assay kit determined the amount of Mg^{2+} released from hydrogels according to the manufacturer's protocol. In brief, 50 μL of standard/sample solution was mixed with 50 μL reaction mix solution and incubated for 30 min at 37 °C. After 10 and 30 min, the absorbance was measured at 450 nm. The concentration of released Mg^{2+} was calculated by using a standard curve (0 – 15 nmol).

To measure the H_2O_2 release behavior, we used the Cu(II)-neocuproine assay as previously reported [2, 3]. We sequentially added 50 μL of 0.01 M phosphate-buffered saline, sample solution, 0.01 M $CuSO_4$, and 0.01 M neocuproine solution and reacted for 20 min. After the reaction, we measured absorbance at 450 nm and calculated the released H_2O_2 concentration from the standard curve (0 – 1000 μM).

Dissolved oxygen (DO) measurement of GtnSH/GtnMI/MgO_2 bioinks

To evaluate the oxygen generation of hydrogels, we monitored the level of DO within hydrogels using a non-invasive oxygen-sensing patch and a commercially used sensor (Presens-4, Regensburg, Germany). Before measuring DO levels, 80 μL of hydrogels was fabricated on the patch sensor in a 96-well plate and we added 160 μL of DPBS after 15 min. We monitored the DO levels at each preset time point (0.5, 1, 3, 6, 9, and 24 h) with 3 s-interval between each point under room temperature condition.

Fourier-transform infrared spectroscopy (FT-IR) and thermogravimetric analyzers (TGA) measurement

The elemental compositions of hydrogel with 0, 10, 20, and 30 μg mL^{-1} of MgO_2 were assessed by FT-IR spectroscopy. The FT-IR spectra were collected using an FT-IR spectrometer (Nicolet IS50, Nicolet Co., Madison, WI), and all spectra were recorded in the transmittance mode within the wavelength range of 4,000 – 500 cm^{-1} by accumulating 100 scans with a resolution of 32 cm^{-1}. The thermal stability of GtnSH/GtnMI/MgO_2 bioink was evaluated by TGA (DSC 8000, Perkin Elmer, Waltham, MA). The initial state of the hydrogel was weighed and heated from RT to 500 ℃ at a heating rate of 10 ℃ min^{-1} in N_2.

In vitro degradation test

To assess the degradability of our hydrogels composed of functionalized gelatin backbone. We fabricated Mg^{2+}-incorporated hydrogels (100 μL) in microtubes and immersed them in 200 μL of DPBS or collagenase solution (5 μg mL^{-1}) at 37 ℃. After removing the solution, the remaining hydrogels (W_d) in microtubes were weighted at predetermined time points (0.5, 1, 3, 6, 9, 24, and 48 h), and fresh solutions were added to the microtubes. The following equation calculated the weight of the degraded hydrogels:

$$\text{Weight of remaining hydrogel (\%)} = (W_d/W_i) \times 100$$

Where W_i is the initial weight of the hydrogel, and W_d is the weight of the remaining hydrogels.

Scanning electron microscopy/energy dispersive x-ray spectroscopy (SEM/EDS)

To investigate the inner pore structure and elemental composition of the hydrogels, we performed SEM/EDS analysis using a scanning electron microscope (JSM 7800F, JEOL, Tokyo, Japan). To prepare the samples for SEM/EDS analysis, we fabricated 100 μL of the hydrogel discs in 1 mL syringe, cut, and freeze-dried samples. Hydrogel disks were incubated in DPBS, which was changed to fresh media every 0.5, 1, 3, 6, 9, 24, 72, 120, and 168 h. After lyophilization, samples were sputter-coated with gold, and elemental composition was measured.

Evaluation of 3D printability

To assess the 3D printing fidelity, a standard printability evaluation method was utilized as per the following equations [5].

$$\mathrm{C} = \frac{4\pi A}{L^2}$$

$$\mathrm{Pr} = \frac{\pi}{4} * \frac{1}{C} = \frac{L^2}{16A}$$

The variables are denoted as follows: C (circularity of lattice squares), L (perimeter of lattice squares), A (area of lattice square), and Pr (printability).

Cell viability test on tissue culture plastic (TCP)-cultured C2C12 cells

To assess the cytocompatibility of MgO_2 particles, the 1.5×10^4 cells mL^{-1} C2C12 cells were seeded on the 96-well plates. After 24 h of incubation, 0 – 1000 μg mL^{-1} of MgO_2 particles were treated on the cells and incubated for 24 h. The Cell Counting Kit-8 (CCK-8) assay solution (diluted in 1:9 v/v in GM, Dojindo, Kumamoto, Japan) was treated on the cultured

cells and reacted for 2 h in a humidified 37 ℃ incubator. The 100 μL of supernatants were moved to new 96-well plates, and their optical densities at 450 nm absorbance were evaluated using the microplate reader (Varioskan Lux, Thermofisher Scientific). For the proliferation assay, the CCK-8 assay was repeated after 1, 3, 5, and 7 d of incubation using the same protocols. Cell membrane integrity was evaluated using Lactate dehydrogenase (LDH) assays (Takara Bio, Shiga, Japan). After 24 h and 48 h of culture, 100 μL of supernatant was transferred to a new 96-well plate. The LDH solution (prepared per the manufacturer's protocol) was added to each well and then incubated for 30 min at RT in the dark. The absorbance was measured at 490 nm using a microplate reader (Varioskan Lux). Cellular oxidative stress induced by MgO_2 was quantified by measuring intracellular reactive oxygen species (ROS) generation using a dichlorofluorescein diacetate (CM-H_2DCFDA) molecular probe (Thermofisher Scientific). The 1.5×10^4 cells well^{-1} C2C12 cells were seeded on the slide glasses and treated with various 0 – 1000 μg mL^{-1} MgO_2 for 24 h. The 1 mM H_2O_2 was treated as a positive control. Subsequently, 5 μM DCFDA solution reacted for 30 min at 37 ℃ dark. Fluorescence microscopy were captured using a confocal laser scanning microscope (CLSM, LSM 800, Carl Zeiss, Oberkochen, Germany) and the fluorescence intensity was measured using a microplate reader.

Immunocytochemical analysis on TCP-cultured C2C12 cells

After 3 d and 7 d of incubation, immunocytochemical analysis was conducted to assess the morphology and myogenic differentiation of C2C12 cells. Based on cytotoxicity analysis, four groups were prepared: MG10, MG20, and MG30 (details in **Table S1**). The cells were washed one to three times with sterilized DPBS between each step. The cells were fixed with 10% formaldehyde (Sigma-Aldrich) for 10 min, followed by permeabilization with 0.1% Triton X-

100 solution for 5 min, and blocking with 2% bovine serum albumin (BSA) solution for 30 min. The anti-myosin heavy chain (MHC) antibodies (Abcam, Cambridge, UK) were added and reacted overnight (12 h) at 4 ℃. On the following day, the samples were treated with fluorescein isothiocyanate (FITC)-labeled goat anti-mouse IgG antibody (Abcam), 165 nM tetramethylrhodamine (TRITC)-labeled phalloidin, and 300 nM 4′,6-diamidino-2-phenylindole (DAPI) solution for 1 h in the dark RT. Fluorescence images were captured using a fluorescence microscope (IX81, Olympus, Tokyo, Japan) or CLSM and analyzed using ZEN software (Carl Zeiss) and ImageJ software.

Immunocytochemical analysis on 3D bioprinted muscle mimetics

The viability of C2C12 cells within the 3D bioprinted constructs was evaluated using a fluorometric live/dead assay. After 1, 3, and 5 d of culture, the muscle mimetics were reacted with 2 μM calcein acetoxymethyl ester (AM) (Thermofisher Scientific) and 4 μM ethidium homodimer-1 (Thermofisher Scientific) for 30 min in the dark incubator at 37 ℃. The stained samples were washed with DPBS and submerged in fresh DPBS to be observed using a CLSM (LSM 800) and quantified using ImageJ software. As described in the previous section, the 3D bioprinted muscle mimetics were treated with 10% formaldehyde for 20 min, 0.1% Triton X-100 solution for 10 min, and 2% BSA solution for 1 h. Subsequently, anti-MHC (Abcam), anti-dystrophin (Abcam), and anti-desmin (Abcam) antibodies were added and reacted overnight at 4 ℃. The following day, the samples were reacted with FITC-labeled goat anti-mouse IgG antibody, 165 nM TRITC-labeled phalloidin, and 300 nM DAPI for 1 h in the dark RT. The stained samples were washed with DPBS and submerged in fresh DPBS to be observed using a CLSM (LSM 800). The myotube length within was calculated using ImageJ software. The fusion index is determined by dividing the number of nuclei within MHC-positive myotubes

by the total number of nuclei observed. Additionally, the maturation index represents the percentage of myotubes containing five or more nuclei within a single myotube.

Western blotting on 3D bioprinted muscle mimetics

After 7 and 14 d of incubation, the C2C12 cells within the 3D bioprinted muscle mimetics were cultivated by digestion of hydrogel with collagenase (Thermofisher Scientific) and then centrifugated. Subsequently, the C2C12 cells were washed with cold DPBS and resuspended in ice-cold RIPA lysis buffer (Santa Cruz Biotechnology, Dallas, TX) for 15 min at 25 ℃. The cell lysates were combined with protein loading buffer (iNtRON Biotechnology, Seongnam, Republic of Korea) for sodium dodecyl sulfate-polyacrylamide gel electrophoresis (SDS-PAGE). The proteins were separated on a 10% SDS-PAGE gel and transferred to a polyvinylidene fluoride membrane (Bio-Rad Laboratories, Hercules, CA). The membrane was blocked with a blocking buffer (Nacalai Tesque, Kyoto, Japan) for 1 h at 25 ℃, followed by overnight incubation with primary antibodies at 4 ℃. Afterward, the membrane was incubated for 1 h with horseradish peroxidase (HRP)-conjugated secondary antibodies (Santa Cruz Biotechnology) at a 1:2000 dilution, and the blots were visualized using a chemiluminescence assay kit (Chemi-Lumi One, Nacalai Tesque Inc.). Primary antibodies used for immunoblotting included MyoD (Abcam), MHC I (Abcam), MHC III (Abcam), Atrogin-1 (Abcam), Muscle-specific RING finger protein 1 (MuRF1, Abcam), peroxisome proliferator-activated receptor gamma coactivator (PGC) 1α (Abcam), and β-actin (Abcam). The data were quantified using ImageJ software and expressed as fold change relative to the control values.

In vivo tissue compatibility test

To demonstrate the tissue compatibility of Mg^{2+}-incorporated hydrogels, we performed *in vivo* mouse subcutaneous implantation. The mice (6-wk-old, female, C57BL/6N) were prepared for this experiment and raised in cages at a standard temperature for 1 and 4 wk. Hydrogel discs (100 μL) were fabricated in 1 mL syringes and subcutaneously implanted in the back of mice in each group: control (hydrogel nontreated group), MG0, MG10, MG20, and MG30. After 7 d and 28 d (hydrogels were completely degraded), mice were sacrificed, and remained hydrogels with skin tissue and the major organs (heart, liver, spleen, lung, and kidney) were harvested. All the collected organs were fixed in formalin solution (neutral buffered, 10%) for 7 d and embedded in paraffin after dehydration in ethanol and xylene. Specimens were sectioned into 3 μm slices and stained with hematoxylin and eosin (H&E). The pathological differences compared to normal tissue (control group) were observed, and the digital images were taken using a light microscope. To evaluate the initial inflammation from implanted hydrogels, sectioned specimens were stained with H&E and immunohistochemical staining for representative marker of macrophages. The sectioned tissue/hydrogels were stained using rabbit specific horseradish peroxidase/3,3'-diaminobenzidine tetrahydrochloride salt (HRP/DAB) detection kit (Abcam) with mouse anti-rabbit polyclonal antibodies of anti-F4/80 (1:100, GeneTex, Irvine, CA) to investigate the macrophage infiltration, according to the manufacturer's instructions.

Immunohistochemical analysis and grip test

To examine muscle histology, 10 μm-thick paraffin-embedded tissue sections were prepared and stained with H&E solution (Abcam) according to the manufacturer's instructions. For immunohistochemistry, the tissue sections were incubated overnight at 4 °C with a polyclonal antibody against MyoG, CD163, CD80, CD4, and CD8 (all Abcam, 1:100 dilution).

Immunoreactions were detected using the EnVision Detection System Kit (Dako, Agilent, Santa Clara, CA), and the nuclei were counterstained with Mayer’s hematoxylin solution. Following staining, images were captured with an AxioScan Z1 digital slide scanner (Carl Zeiss) and analyzed using Zen software. Quantitative data such as injured area, muscle mass, fiber diameter, and the number of inflammatory cells were obtained from the H&E images using ImageJ software.

The grip strength of the mice's hindlimbs was measured using a grip strength meter equipped with a pull bar. To assess grip strength, the mice's paws were placed on the pull bar and gently pulled until the hindlimb released the bar (Bioseb, Virtolles CEDEX, France) after a predetermined time following the transplantation of GtnSH/GtnMI/MgO_2 constructs. The maximum grip strength before release was recorded.

Statistical analysis

All variables were assessed in triplicate, with the experiment repeated twice (n = 6). The results are expressed as the mean ± standard deviation (SD). Before conducting statistical analysis, the data were evaluated for homogeneity of variance using Levene’s test. Statistical comparisons were made using one-way analysis of variance (ANOVA) or Student’s t-test. The levels of statistical significance were designated as follows: *$p < 0.05$, **$p < 0.01$, ***$p < 0.001$, ****$p < 0.0001$.

Table S1. Sample codes and detailed concentrations for test groups.

**Group codes**	**GtnSH (w/v%)**	**GtnMI (w/v%)**	**MgO_2 (μg mL^{-1})**
MG0	3	3	-
MG10	3	3	10
MG20	3	3	20
MG30	3	3	30

Table S2. Comparison of Mg^{2+} ratio between 0 d and 7 d.

**0 d**	**0**	**10**	**20**	**30**
C	47.13	49.04	46.69	41.92
N	20.34	19.78	20.22	20.06
O	32.38	31.01	32.87	37.64
Mg	0.15	0.17	0.22	0.38

**7 d**	**0**	**10**	**20**	**30**
C	46.3	45.98	47.13	47.48
N	18.98	20.05	20.39	18.01
O	34.58	33.81	32.28	34.14
Mg	0.14	0.16	0.2	0.37

Table S3. Comparison of myogenic and immune modulating effects between GtnSH/GtnMI/MgO_2 bioink and previously reported bioinks. Abbreviations: reactive oxygen species, ROS; porcine decellularized skeletal muscle extracellular matrix, mdECM; α-bungarotoxin, BTX; poly-3,4-ethylene dioxythiophene, PEDOT; gelatin methacrylate, GelMA; decellularized extracellular matrix, dECM; nuclear β-galactosidase, nLacZ; mesoangioblast, Mabs, insulin-like growth factor-1, IGF-1; mesenchymal stem cell, MSC; human umbilical vein endothelial cell, HUVEC.

Components	Cell viability and growth	*In vitro* myogenesis	*In vivo* myogenesis and immune modulation	Refs.
GtnSH/GtnMI/MgO_2	- Mostly viable<br>- Enhanced proliferation and F-actin spreading<br>- No membrane damage and intracellular ROS	- Modulation of myogenic markers (MHC, MyoD, Atrogin-1, MURF1)<br>- Dystrophin, desmin, MHC expression<br>- Increased myogenesis indexes	- Lowered T cell activity<br>- M2 macrophage polarization<br>- Muscle tissue regeneration<br>- Grip strength	This study
mdECM	- Mostly viable<br>- Oriented behavior	- MHC and BTX expression	NA	[6]
GelMA/PEDOT	- Mostly viable<br>- F-actin spreading	- MyoG and desmin expression	NA	[7]
dECM/Gelatin	- Mostly viable<br>- Highly aligned F-actin spreading<br>- Enhanced proliferation<br>- Vasculogenic gene expression	- MHC<br>- Myotube index<br>- Myogenic gene expression	- Human nuclei in transplantation site<br>- Isometric torque and contraction<br>- Muscle tissue regeneration<br>- Vascularization<br>- Neuromuscular junction	[8]
Pegylated fibrinogen with nLacZ-Mabs	- Mostly viable<br>- Aligned F-actin spreading	- MHC, desmin, and laminin expression<br>- Myofibril formation and indexes	- Vascularization<br>- Muscle tissue regeneration<br>- Innervation and stem cell niche	[9]

IGF-1/GelMA/gelatin	Proliferation and F-actin spreading	Myogenic markers (MRF1, Collagen, β1-integrin, and α-actinin)	- Torque and tetanus strength<br>- Laminin, acetylcholine, MHC, and MF20<br>- Enhanced running distance	[10]
Fibrin/Matrigel	NA	- Phosphoproteome analysis showing muscle maturation, axonogenesis, and angiogenesis	- Optogenetic ion channel and MHC<br>- Muscle tissue regeneration<br>- Enhanced exercise with response speed<br>- Phosphoproteome analysis	[11]
Collagen/MSC/HUVEC	- F-actin spreading and proliferation	- Prevascularization	- Enhanced muscle regeneration<br>- MHC, laminin, and MF20<br>- Muscle fiber indexes and vascularization<br>- Enhanced motor functions<br>- Neuromuscular junction formation	[12]

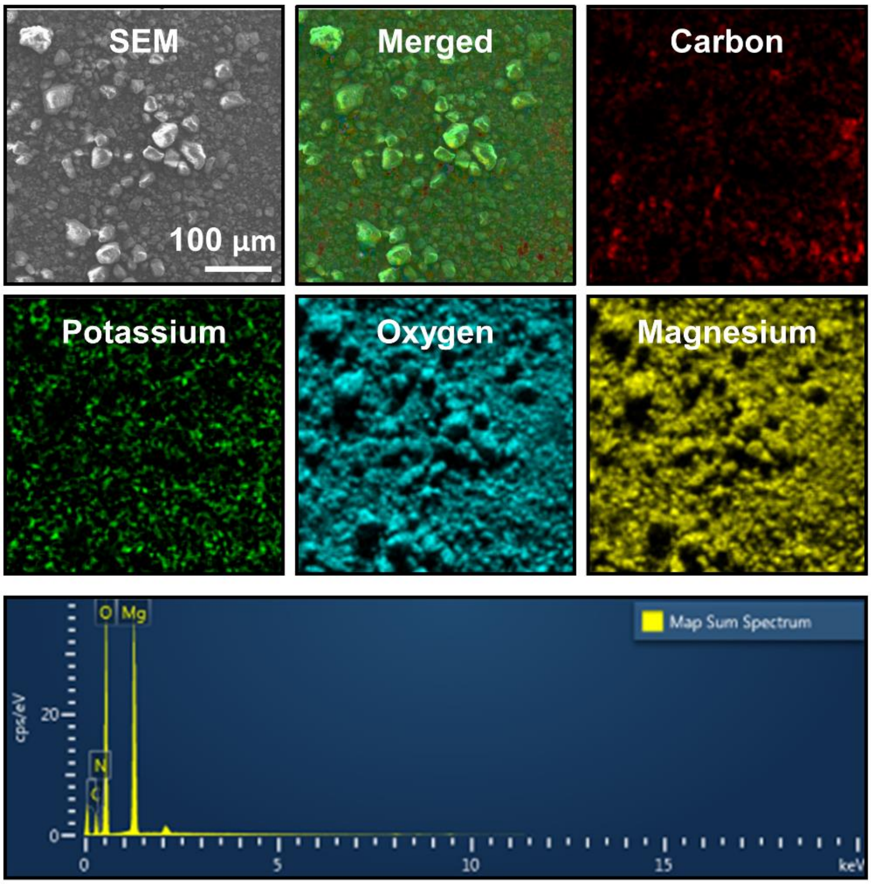

Figure S1. SEM/EDS of MgO_2 particles. A scale bar indicates 100 µm.

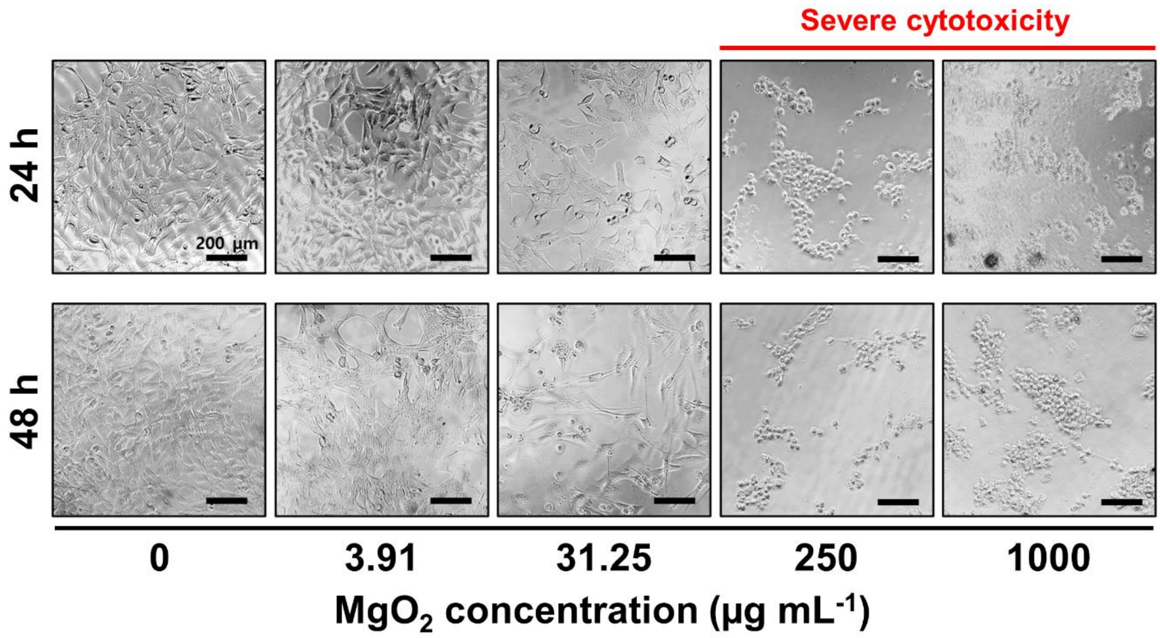

Figure S2. Cytotoxicity of MgO_2 particles on C2C12 cells cultured on TCP after 24 h and 48 h. Images were captured by optical microscope. Scale bars indicate 200 µm.

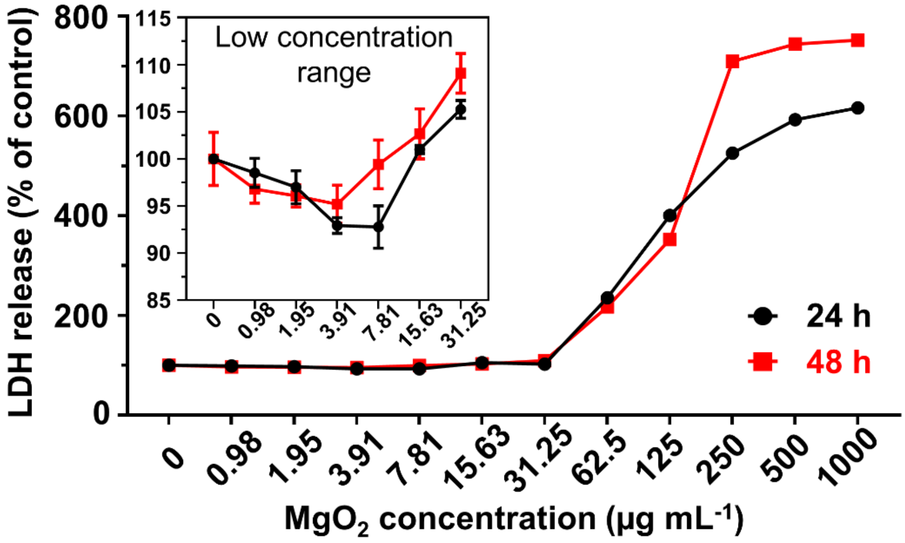

Figure S3. LDH release from MgO_2 particles-treated C2C12 cells cultured on TCP after 24 h and 48 h. The inset graph denotes the low concentration range.

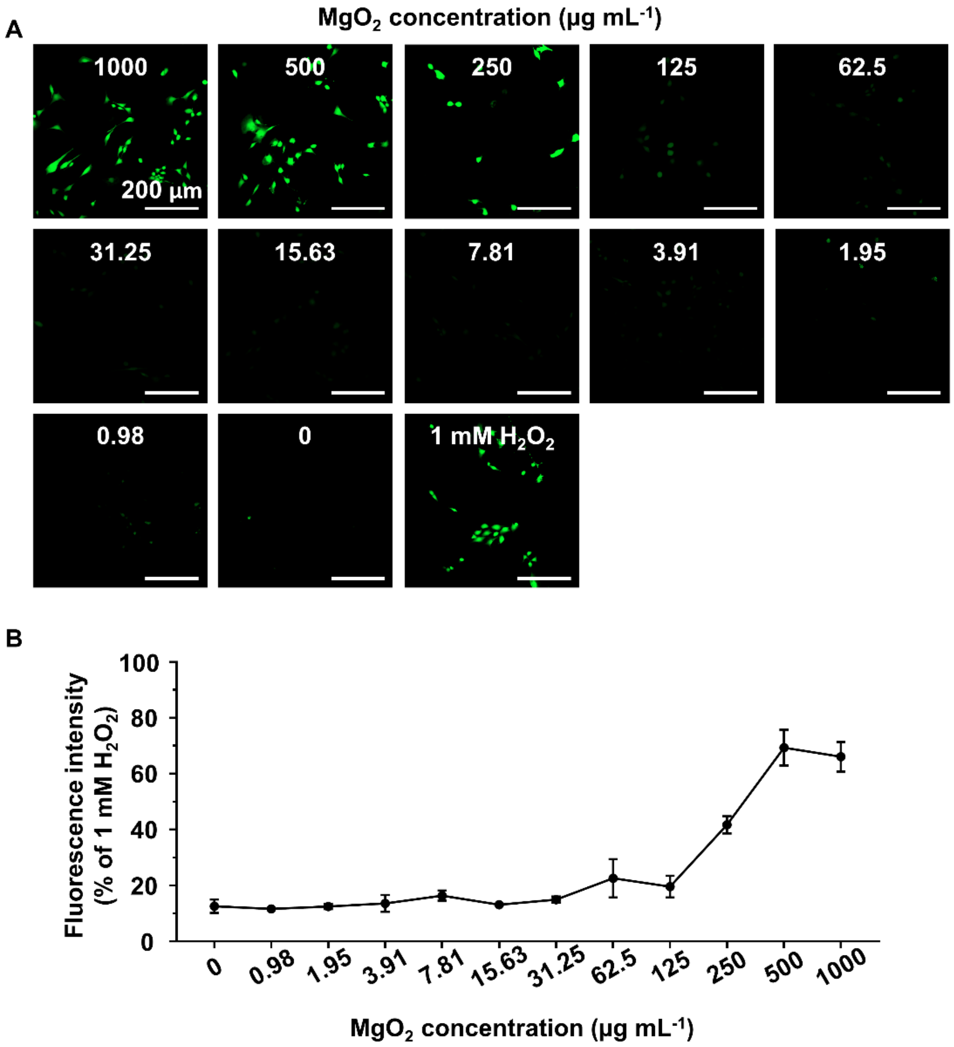

Figure S4. DCFDA assay for detection of intracellular ROS generation induced by MgO_2. (A) DCFDA fluorescence images and (B) relative fluorescence intensity. Scale bars indicate 200 μm.

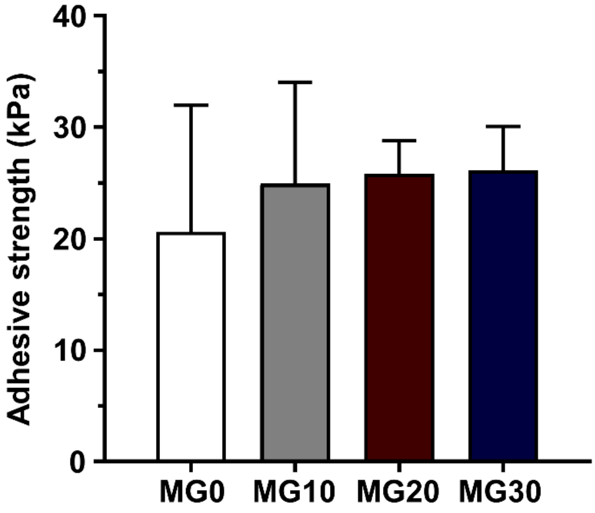

Figure S5. Adhesive strength for GtnSH/GtnMI/MgO_2 hydrogels on decellularized porcine skins.

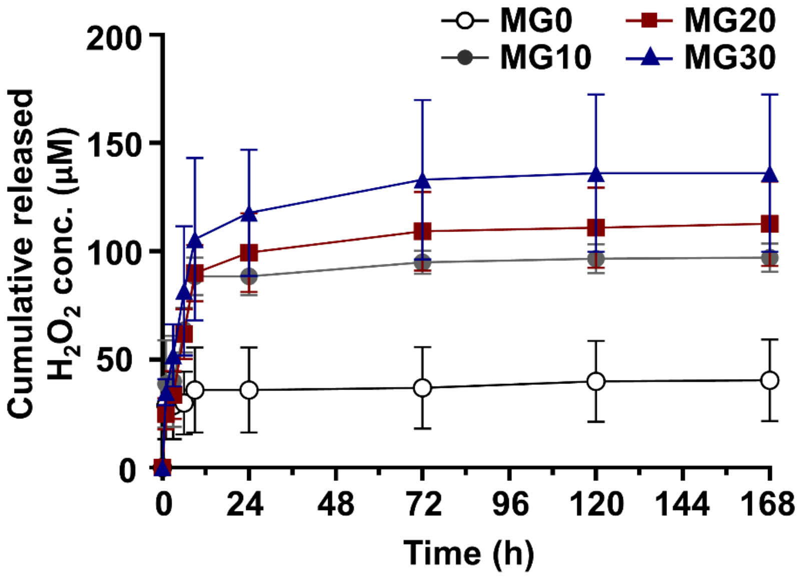

Figure S6. Quantified H_2O_2 release from GtnSH/GtnMI/MgO_2 hydrogels for 168 h (7 d) *in vitro*.

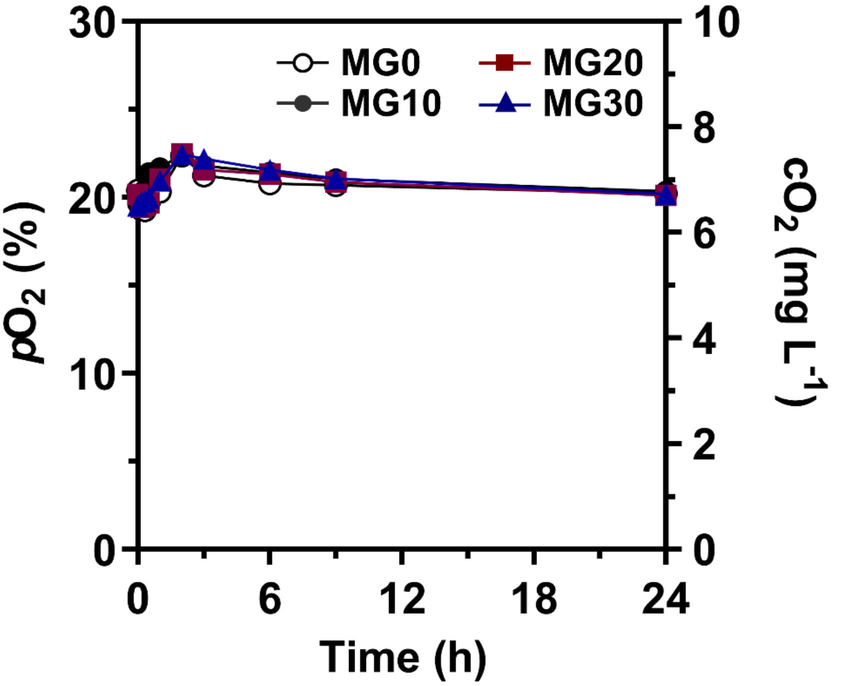

Figure S7. DO level measurement at the bottom of hydrogel to investigate the O_2 generation.

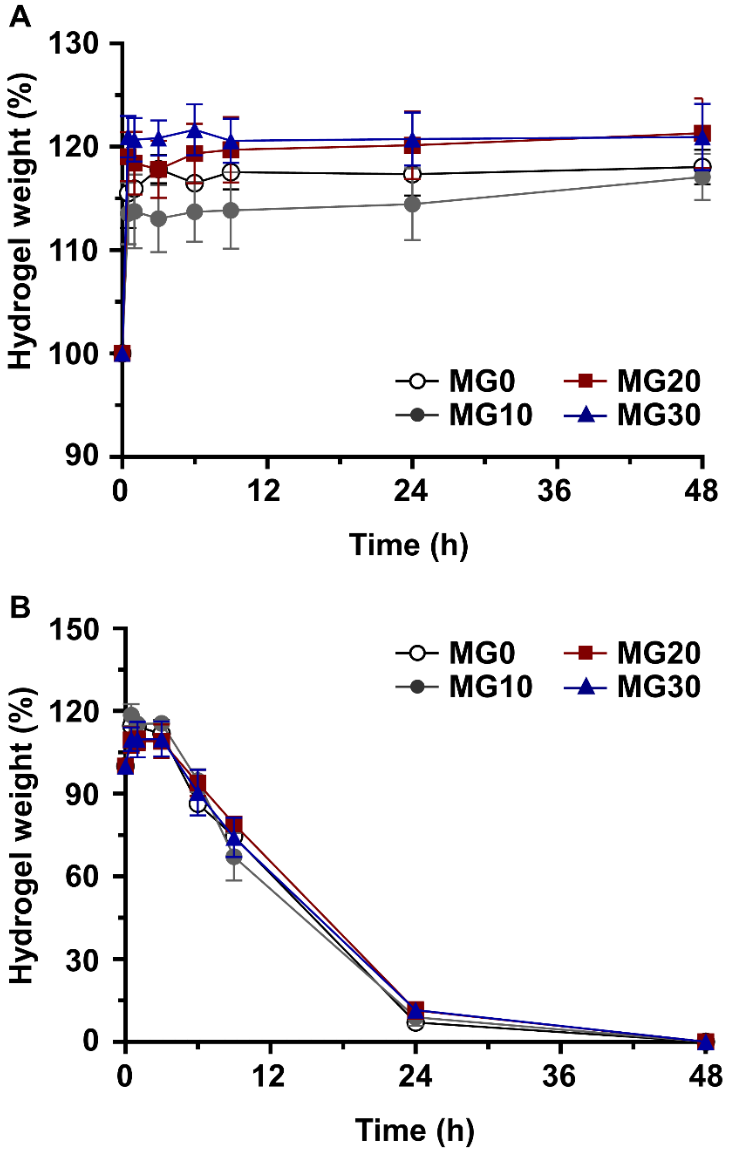

Figure S8. *In vitro* degradation of GtnSH/GtnMI/MgO_2 hydrogels in (A) DPBS and (B) 5 μg mL^{-1} collagenase for 48 h.

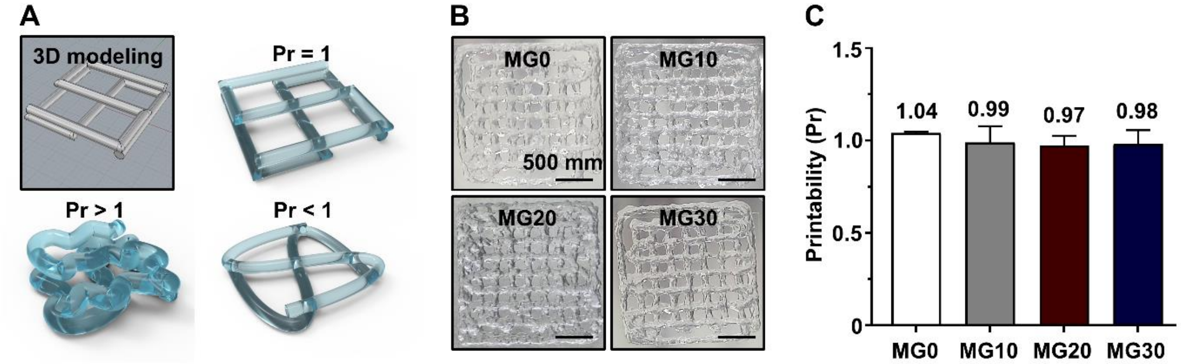

Figure S9. 3D printability of GtnSH/GtnMI/MgO_2 bioinks. (A) Rendered image and schematic diagram of printed constructs at different Pr values. (B) Digital images of printed constructs. (C) Quantified Pr according to the different MgO_2 concentrations. Scale bars indicate 500 mm.

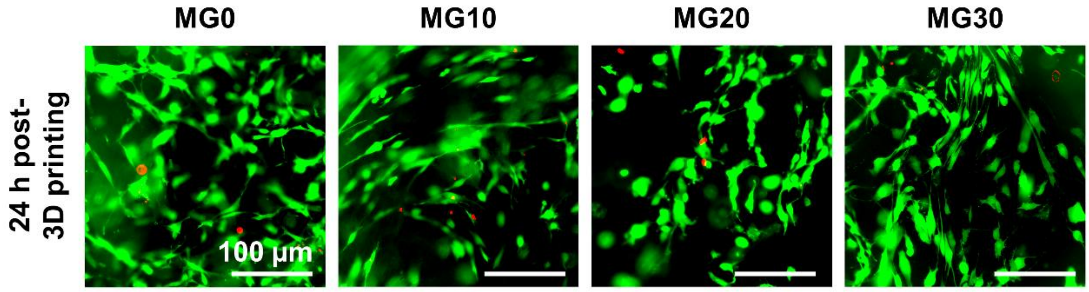

Figure S10. Live/dead assay after 24 h of 3D printing. Live cells are stained with green (calcein AM), while dead cells are stained with red (ethidium homodimer-1). Scale bars indicate 100 µm.

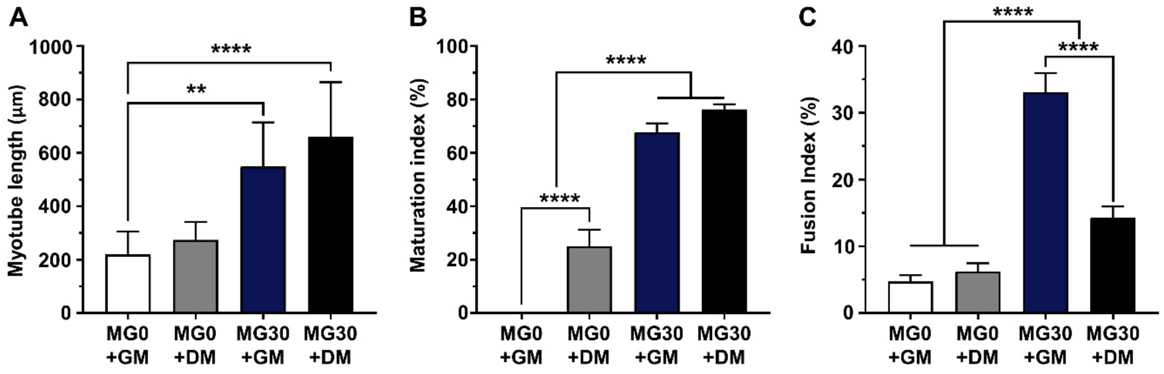

Figure S11. Quantification of differentiation indices after 7 d. (A) Myotube length, (B) maturation index, and (C) fusion index. Asterisks indicate significant differences between groups (**p < 0.01 and ****p < 0.0001)

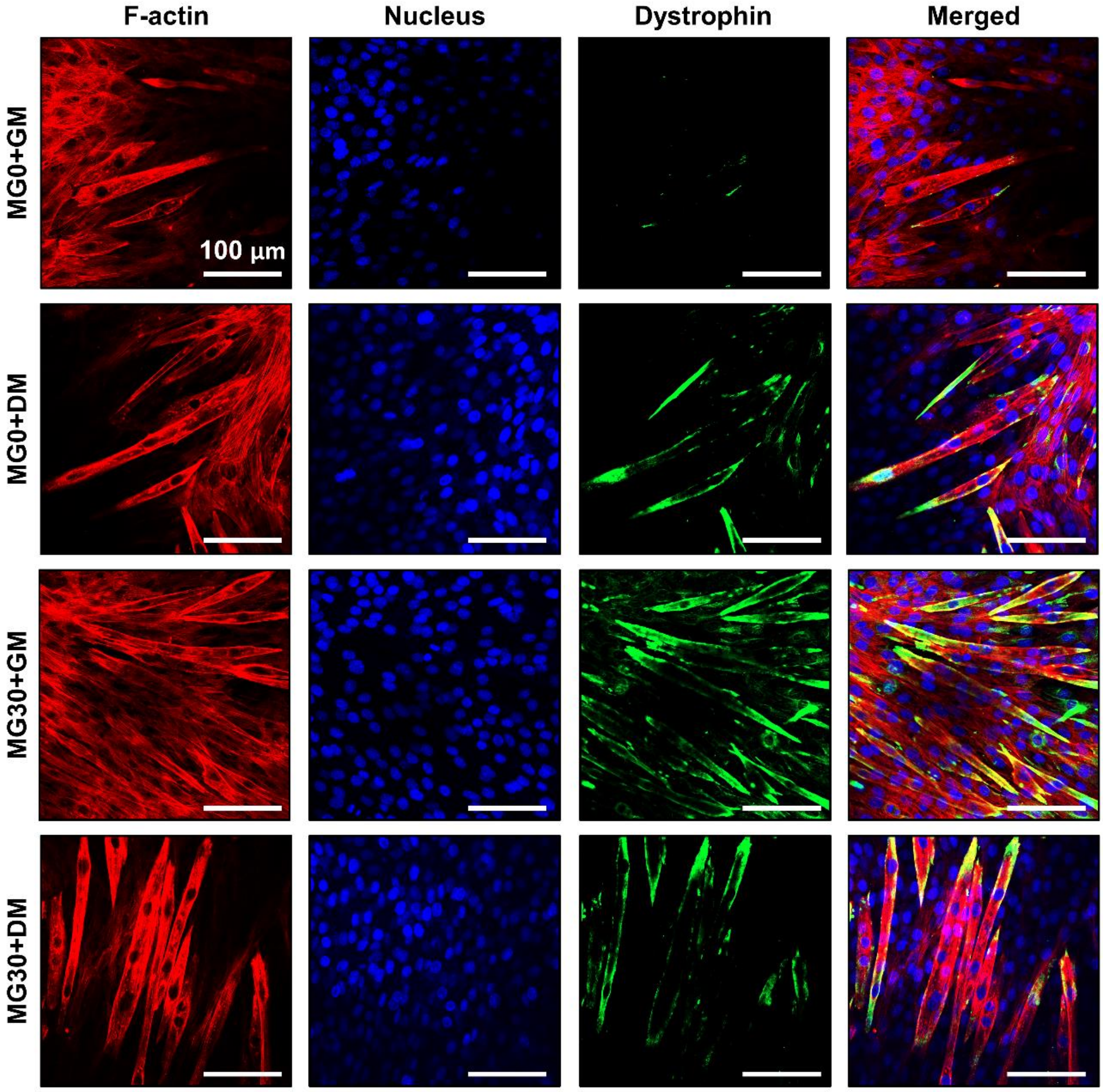

Figure S12. Immunofluorescence staining of 3D bioprinted constructs after 7 d. Each fluorescence channel represents the following: TRITC (red) for F-actin, DAPI (blue) for nucleus, and FITC (green) for dystrophin. Scale bars indicate 100 µm.

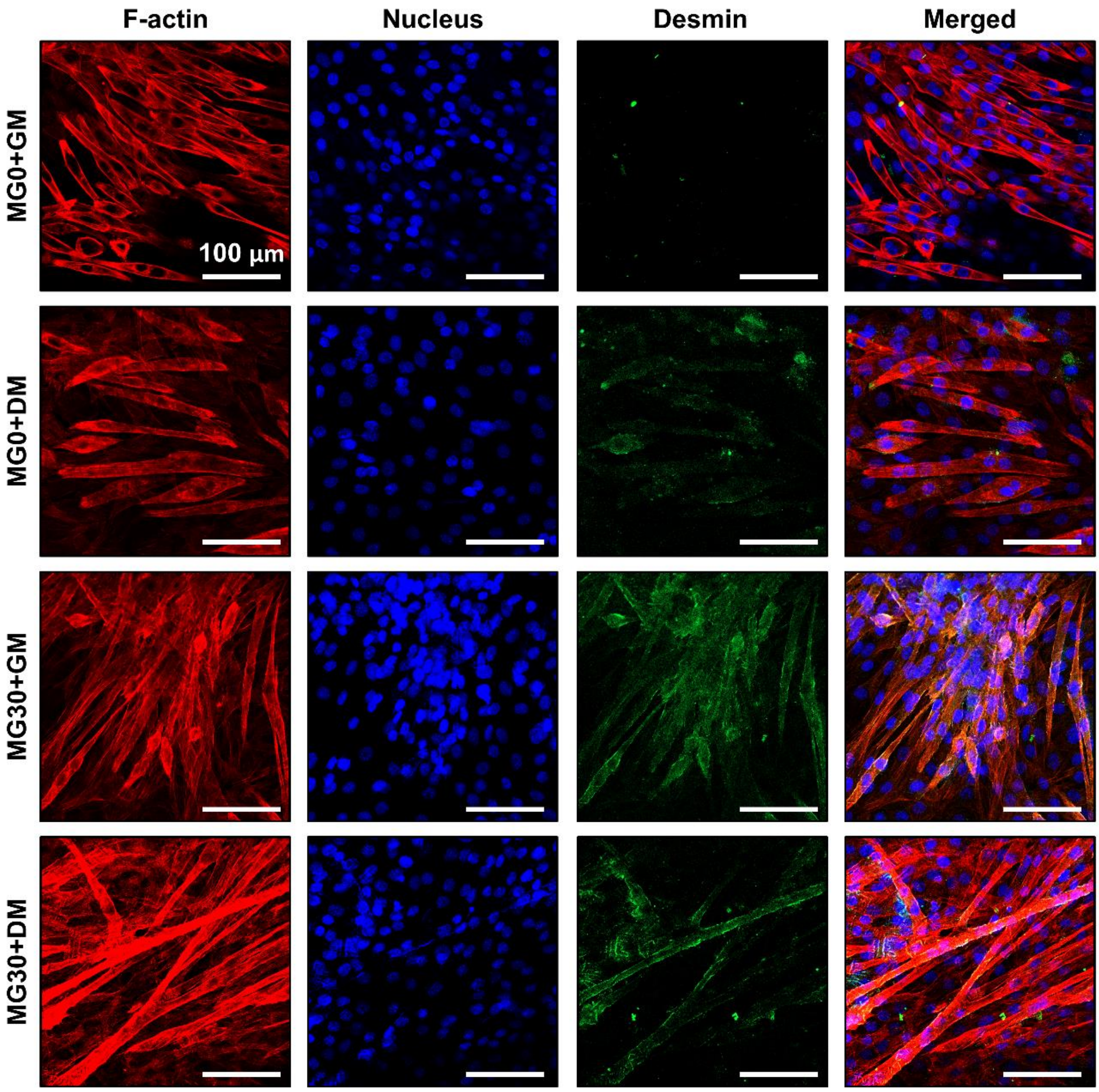

Figure S13. Immunofluorescence staining of 3D bioprinted constructs after 7 d. Each fluorescence channel represents the following: TRITC (red) for F-actin, DAPI (blue) for nucleus, and FITC (green) for desmin. Scale bars indicate 100 μm.

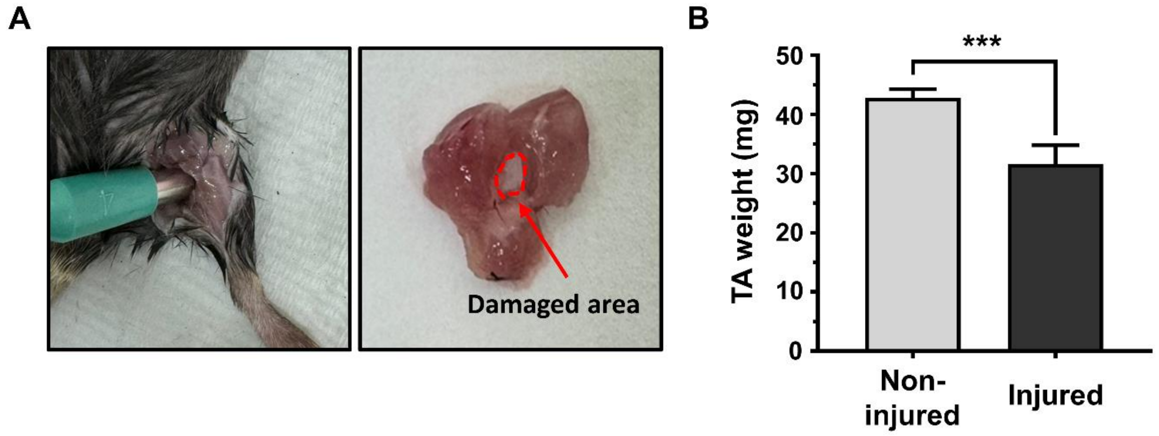

Figure S14. Induction of VML on mouse TA muscles using a 2 mm diameter biopsy punch. (A) Digital images and (B) weight of TA muscles before and after surgical injuries. Asterisks indicate significant difference between groups (***$p < 0.001$).

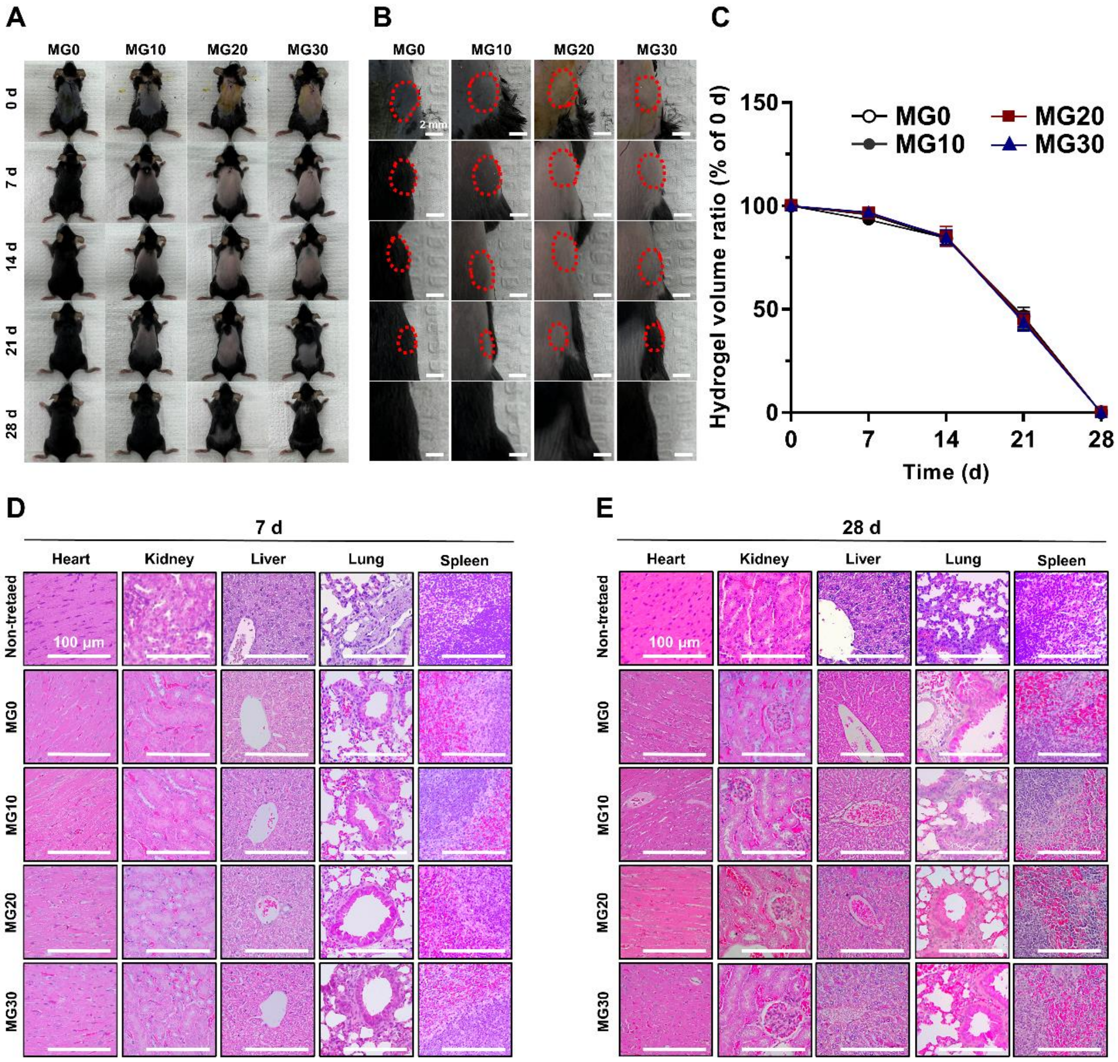

Figure S15. *In vivo* biodegradation and organ compatibility of subcutaneously implanted GtnSH/GtnMI/MgO_2 hydrogels. (A) Representative and (B) magnified digital images. (C) Change of hydrogel volume ratio for 28 d. H&E staining on essential organs after (D) 7 d and (E) 28 d of subcutaneous implantation. Scale bars indicate 2 mm for (B) and 100 μm for (D, E).

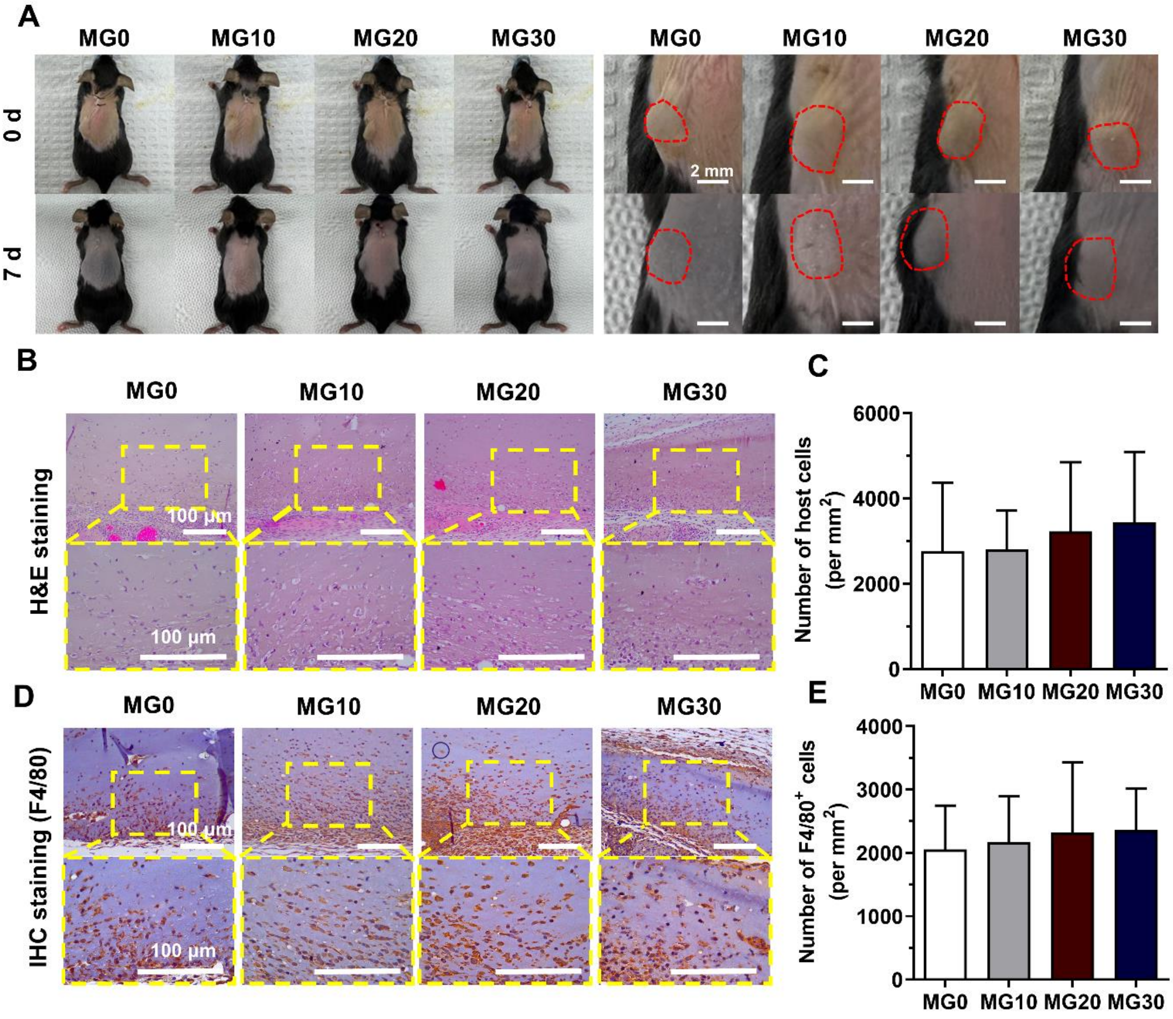

Figure S16. Foreign body reaction after subcutaneous implantation of GtnSH/GtnMI/MgO_2 hydrogels. (A) Representative and magnified digital images. Infiltration of (B, C) host cells and (D, E) F4/80$^+$ cells into the implanted hydrogels on 7 d. Scale bars indicate 2 mm for (A) and 100 µm for (B, D).

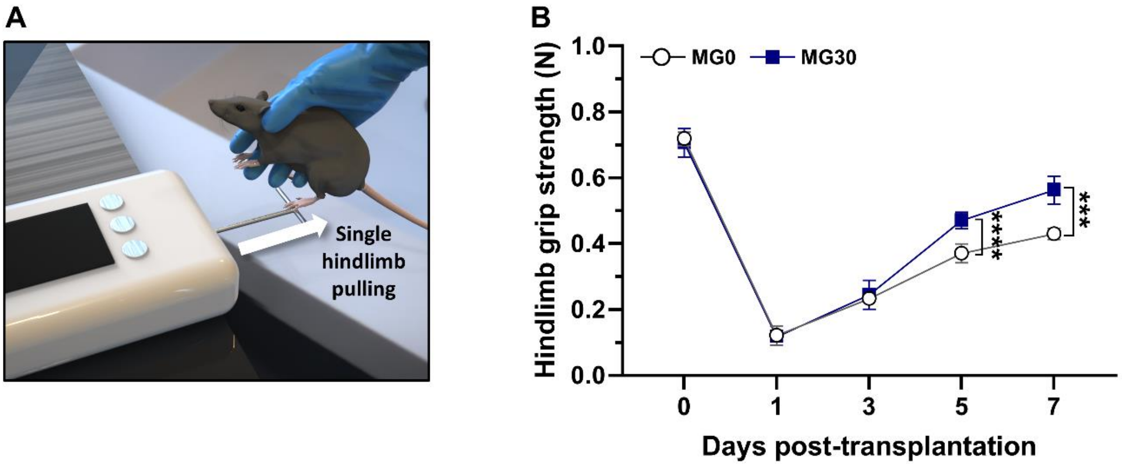

Figure S17. Grip test measuring the strength of a single hindlimb during 7 d post-transplantation of GtnSH/GtnMI/MgO_2 constructs. (A) A schematic illustration of the grip test and (B) the recorded grip strength of the mice. Asterisks indicate significant differences between groups (***$p < 0.001$ and ****$p < 0.0001$).

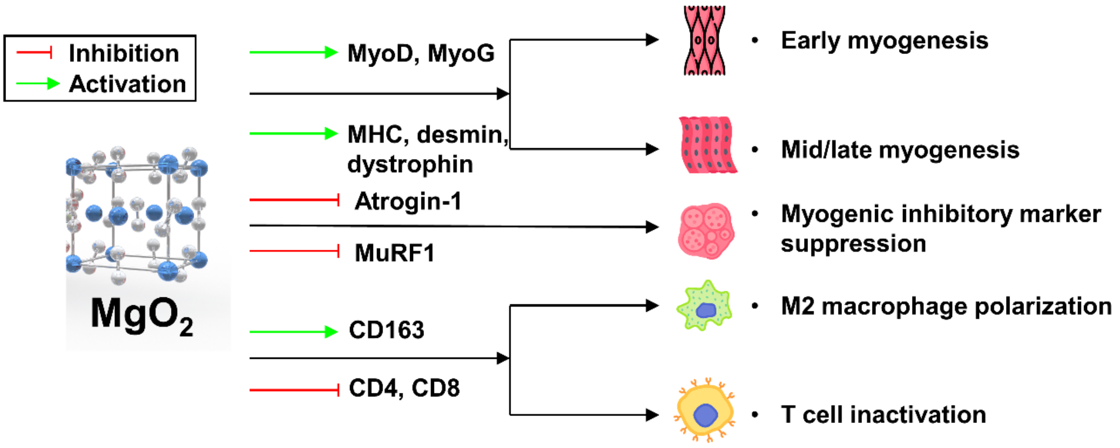

Figure S18. Schematic diagram illustrating the proposed mechanism of MgO_2 in myogenesis.

References

1. Jeon HR, Kang JI, Bhang SH, Park KM, Kim D-I. Transplantation of stem cell spheroid-laden 3-dimensional patches with bioadhesives for the treatment of myocardial infarction. Biomater Res. 2024; 28: 0007.
2. Park S, Park KM. Hyperbaric oxygen-generating hydrogels. Biomaterials. 2018; 182: 234-44.
3. Han MJ, An JA, Kim JM, Heo DN, Kwon IK, Park KM. Calcium peroxide-mediated bioactive hydrogels for enhanced angiogenic paracrine effect and osteoblast proliferation. J Ind Eng Chem. 2023; 120: 121-30.
4. Kang JI, Park KM. Oxygen-supplying syringe to create hyperoxia-inducible hydrogels for in situ tissue regeneration. Biomaterials. 2023; 293: 121943.
5. Ouyang L, Yao R, Zhao Y, Sun W. Effect of bioink properties on printability and cell viability for 3D bioplotting of embryonic stem cells. Biofabrication. 2016; 8: 035020.
6. Choi YJ, Kim TG, Jeong J, Yi HG, Park JW, Hwang W, et al. 3D cell printing of functional skeletal muscle constructs using skeletal muscle-derived bioink. Adv Healthc Mater. 2016; 5: 2636-45.
7. Wang Y, Wang Q, Luo S, Chen Z, Zheng X, Kankala RK, et al. 3D bioprinting of conductive hydrogel for enhanced myogenic differentiation. Regen Biomater. 2021; 8: rbab035.
8. Choi Y-J, Jun Y-J, Kim DY, Yi H-G, Chae S-H, Kang J, et al. A 3D cell printed muscle construct with tissue-derived bioink for the treatment of volumetric muscle loss. Biomaterials. 2019; 206: 160-9.
9. Fornetti E, De Paolis F, Fuoco C, Bernardini S, Giannitelli S, Rainer A, et al. A novel extrusion-based 3D bioprinting system for skeletal muscle tissue engineering. Biofabrication. 2023; 15: 025009.
10. Endo Y, Samandari M, Karvar M, Mostafavi A, Quint J, Rinoldi C, et al. Aerobic exercise and scaffolds with hierarchical porosity synergistically promote functional recovery post volumetric muscle loss. Biomaterials. 2023; 296: 122058.
11. Rousseau E, Raman R, Tamir T, Bu A, Srinivasan S, Lynch N, et al. Actuated tissue engineered muscle grafts restore functional mobility after volumetric muscle loss. Biomaterials. 2023; 302: 122317.
12. Wei SY, Chen PY, Tsai MC, Hsu TL, Hsieh CC, Fan HW, et al. Enhancing the repair of substantial volumetric muscle loss by creating different levels of blood vessel networks using pre-vascularized nerve hydrogel implants. Adv Healthc Mater. 2024; 13: 2303320.
